# Divergent trajectories of Arctic change: Implications for future socio-economic patterns

**DOI:** 10.1007/s13280-024-02080-x

**Published:** 2024-10-30

**Authors:** Abbie Tingstad, Kristin Van Abel, Mia M. Bennett, Isabelle Winston, Lawson W. Brigham, Scott R. Stephenson, Margaret Wilcox, Stephanie Pezard

**Affiliations:** 1 U.S. Coast Guard Academy, 31 Mohegan Ave Pkwy, New London, CT 06320 USA; 2 RAND Corporation, 1776 Main St, Santa Monica, CA 90401 USA; 3 Department of Geography, University of Washington, Smith Hall 408 A, Box 353550, Seattle, WA 98195-3550 USA; 4 UAF Troth Yeddha’ Campus, Syun-Ichi Akasofu Building, PO Box 757340, Fairbanks, AK 99775-7340 USA

**Keywords:** Arctic, Climate change, Delphi method, Forecasting, Governance, Socio-economic change

## Abstract

Climate change is causing rapid warming in the Arctic, which, alongside other physical, socio-economic, cultural, geopolitical, and technological factors, is driving change in the far north. This research presents a conceptual model summarizing Arctic change factors which in turn was used in the design of a Delphi exercise which leveraged a variety of experts to forecast trajectories in different parts of the Arctic. Based on these experts’ expectations for economic and governance outcomes by 2050, we find that our results illustrate the “many Arctics” concept or some of the ways in which the Arctic is heterogenous now, and perhaps becoming increasingly so in the future. Sub-regions of the Arctic differed in expert expectations about the future of resource extraction, tourism, Indigenous self-determination, and military activity, among other outcomes. This work also discusses the post-2022 geopolitical situation and some potential implications of “many Arctics” for policy and future governance.

**Supplementary Information:**

The online version contains supplementary material available at 10.1007/s13280-024-02080-x.

## Introduction

The Arctic has been warming at nearly four times the global average since 1979 (Rantanen et al. 2022) and is an increasingly clear example of how physical and social climate change impacts are unfolding (e.g., Li et al. 2021; Brigham and Gamble 2022; Streletskiy et al. 2023; Ayeb-Karlsson et al. 2024; Gillis et al. 2024; Ksenofontov and Petrov 2024; Stokke 2024; and Wang 2024). A body of work documents ongoing and projected changes to first-year and multi-year sea ice (e.g., Wunderling et al. 2020; Dai and Jenkins 2023), permafrost (e.g., Streletskiy et al. 2023), viability of transportation infrastructure (e.g., Dong et al. 2022; Fatolahzadeh Gheysari and Maghoul 2024), coastal erosion (e.g., Nielsen et al. 2022), ecosystem shifts (e.g., Huntington et al. 2020; Madani et al. 2023), and increasingly inhospitable landscapes for human occupation and use (e.g., Hori et al. 2018; Ramage et al. 2021). These physical impacts are not uniform across the region and reflect variability in geography, geology, and weather patterns. For instance, the Nordic Arctic has a more temperate climate caused by the Gulf Stream (e.g., Asbjørnsen et al. 2023) and is thus relatively densely populated, farmed, and urbanized as compared with many other places in the Arctic such as Siberia, Alaska, and northern Canada.

Across the maritime Arctic, seasonal access via ship and boat is generally growing due to sea ice loss. Yet this same effect, by making sea ice thinner and more fragile, is reducing access by vehicles that rely on stable ice, such as snowmobiles. In turn subsistence hunting can be riskier (e.g., City of Savoonga 2023) and reduced mobility impacts coastal communities in places like northern Canada otherwise only accessible to each other via air. In addition, many inland and coastal areas are becoming harder to access due to warming, accelerating coastal erosion and impacting year-round and seasonal road stability (e.g., Nielsen et al. 2022). Some northern maritime routes are potentially becoming more hazardous for navigation in the next few decades as Arctic sea ice patterns shift (e.g., Cook et al. 2024).

Similarly, the socio-cultural and economic geography of the Arctic varies considerably across the region, as it has throughout human history. The Arctic Human Development Report (AHDR) first published in 2004 and again in 2014 is a pioneering effort to document the region’s diversity. Business Index North (BIN) (Middleton et al. 2019) seeks to visualize primarily economic complexity through exploring variability in different metrics relevant to the cash economy across the region. Other efforts such as those by Pezard et al. (2017) and Heininen et al. (2020) have examined the landscape of Arctic strategies and cooperative frameworks. Yet others—such as Lavissière et al. (2020) for Arctic transportation—have summarized the geography and academic work on different sectors within the Arctic.

Over the past two decades, several efforts have sought to depict scenarios of social, economic, geopolitical, and technological change in the Arctic, many of them considering regional outcomes several decades into the future. In 2009, the Arctic Marine Shipping Assessment (AMSA) (Brigham 2010; Fretheim et al. 2011) leveraged subject matter experts to identify drivers of change by 2050, ultimately selecting the factors of governance as well as resources/trade as two key axes of change to develop four visions for the Arctic’s maritime future. Other research such as that by Middleton et al. (2021) has considered drivers such as geopolitics and technological change.

Whereas the body of work on projections of future physical Arctic change has been steadily growing, there is considerably less research looking systematically at expectations for parallel socio-economic changes in coming decades. The research presented here seeks to narrow that gap by building on previous social science efforts and integrating knowledge of physical changes to characterize possible socio-economic trajectories by 2050. Finally, while the bulk of the empirics for this study were gathered before 2022, we also consider our findings with respect to the Arctic’s dramatically changed geopolitical environment following Russia’s full-scale invasion of Ukraine. The deepening split between Russia and the West makes analysis of the ways in which the Arctic’s various sub-regions could change all the more pressing to consider, but also necessitates a look at how a fundamental geopolitical shift impacts trajectories of change in the Arctic and the influence of other drivers on these changes.

Our work differs from previous pan-Arctic studies in two distinct ways. First, it considers sub-regions of the Arctic as opposed to the region as a whole, potentially allowing for richer results. Different sub-regions of the Arctic may be trending in different socio-economic directions, just as projected future physical changes are not uniform. Second, this research leverages data from the application of the Delphi expert elicitation methodology to characterize future socio-economic trajectories in each sub-region. This is the first time the Delphi method has been used for the particular topic of forecasting socio-economic trajectories across the pan-Arctic.

## Theoretical framework

**Fig. 1:**
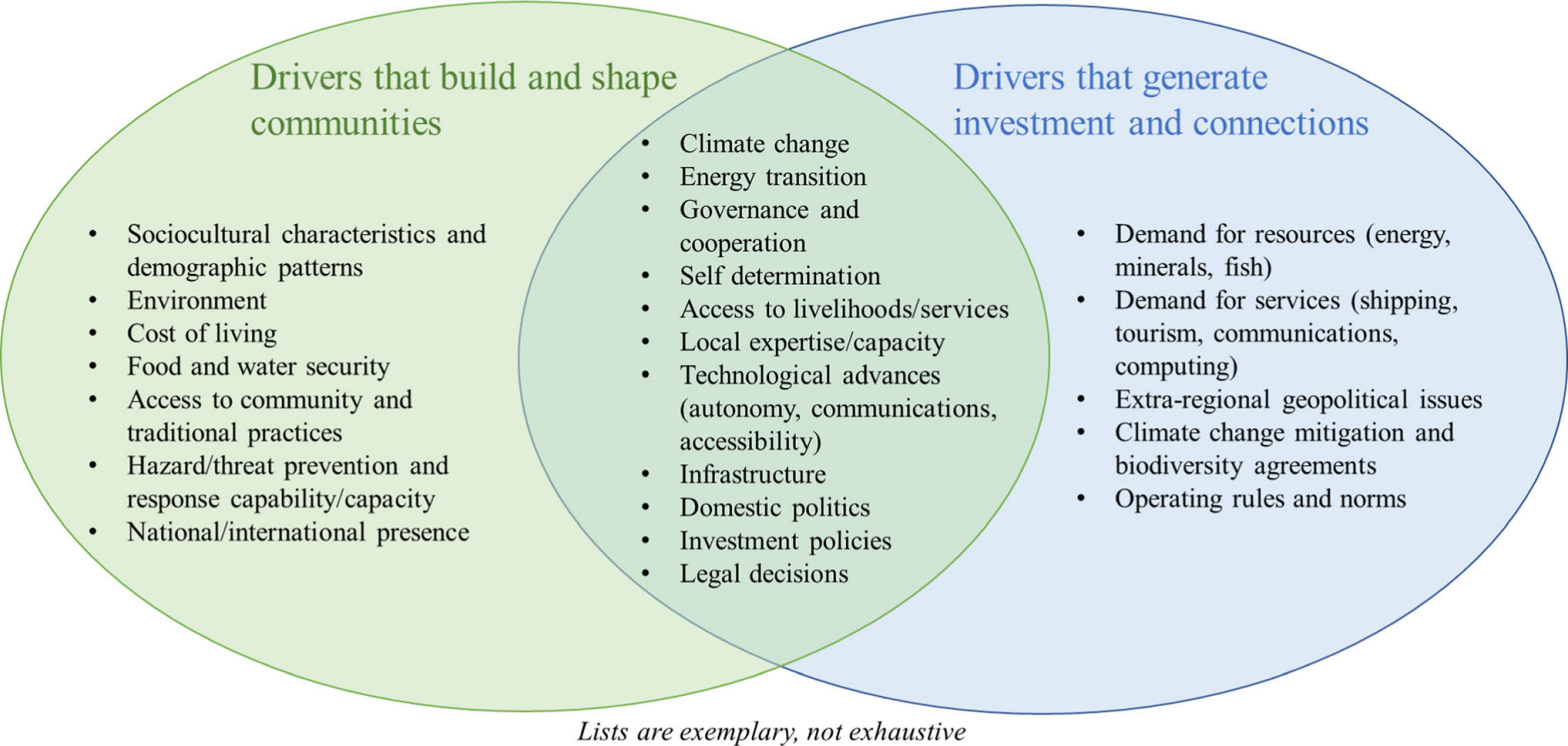
Delineation of selected Arctic change drivers

Figure 1 summarizes the concept for understanding some types of Arctic change drivers that could influence outcomes by 2050. These change drivers revealed from our preliminary research, documented in the next section, can be placed into three categories: drivers that fundamentally shape activities in and characteristics of local communities; those that drive attention toward the pan-Arctic from the outside; and those with impacts at the community level and also drive global interest in the Arctic. The research team believed it was important to consider change drivers from both local (“inside looking out”) and global (“outside looking in”) perspectives. This distinction follows longstanding debates within geographic theory about the mutual importance of and distinction between the local and the global, for example, as *spaces of dependence* in the former case versus *spaces of engagement* in the latter (e.g., Holloway 2008).

In this figure, the change drivers that build and shape communities, such as socio-cultural characteristics and the environment, are inherently local and influence community land and marine use, and how people view the world and their place within it. For example, demographic and cultural patterns in Greenland and Alaska make women more likely to migrate away from small villages to large cities. Although opportunities for hunting and fishing are likely to entice men to stay, women are more likely to pursue career and educational opportunities away from home, particularly university degrees (Hamilton et al. 1996).

Change drivers that generate investments from and connections with the outside are global in nature as they draw the outside world to the Arctic. For example, global demand for resources and services have raised interest in ongoing and potential extractive activities in the far north, which largely reflect economic factors outside the Arctic (Keil 2017), including the rise of China and India as major resource consumers.

Several drivers overlap these categories; in particular, those represented in the center of this diagram such as climate change and governance issues. The impacts of such drivers differ depending on context and scale. The global energy transition is increasing global interest in the Arctic’s critical minerals as well as shaping local tensions over renewable energy projects and the green transition (McCauley et al. 2022). Degrees of Indigenous self-determination also impact trajectories in a variety of areas including subsistence practices and resource and infrastructure development (Nuttall 2019).

## Materials and methods

This research relied on a mixed methods approach to examine expert expectations for certain types of socio-economic change across the Arctic. Results from a literature review (references for which are referred to throughout this article), discussions with subject matter experts recruited to the research project’s International Reference Group^1^ (Converging Pressures on Arctic Development undated), and assumptions-testing were used to feed into a Delphi forecasting exercise, a method of building consensus among experts by using consecutive questionnaires. These methods are described in detail in what follows with additional information provided in the accompanying Supplementary Information.

### Geographic scope

For the purposes of the analyses detailed here, we employed a definition of the Arctic from the 2004 Arctic Human Development Report: “…all of Alaska, Canada North of 60°N together with northern Quebec and Labrador, all of Greenland, the Faroe Islands, and Iceland, and the northernmost counties of Norway, Sweden and Finland…the Murmansk Oblast, the Nenets, Yamalo Nenets, Taimyr, and Chukotka Autonomous Okrugs, Vorkuta City in the Komi Republic, Norilsk and Igarka in Krasnoyarsky Kray, and those parts of the Sakha Republic whose boundaries lie closest to the Arctic Circle” (Arctic Human Development Report 2004, p. 18). Additionally, the research team recognizes all parts of the Arctic Ocean as part of the Arctic, as well as all recognized exclusive economic zones (EEZs) along the coasts of the land areas described in the Arctic Human Development Report.

**Fig. 2:**
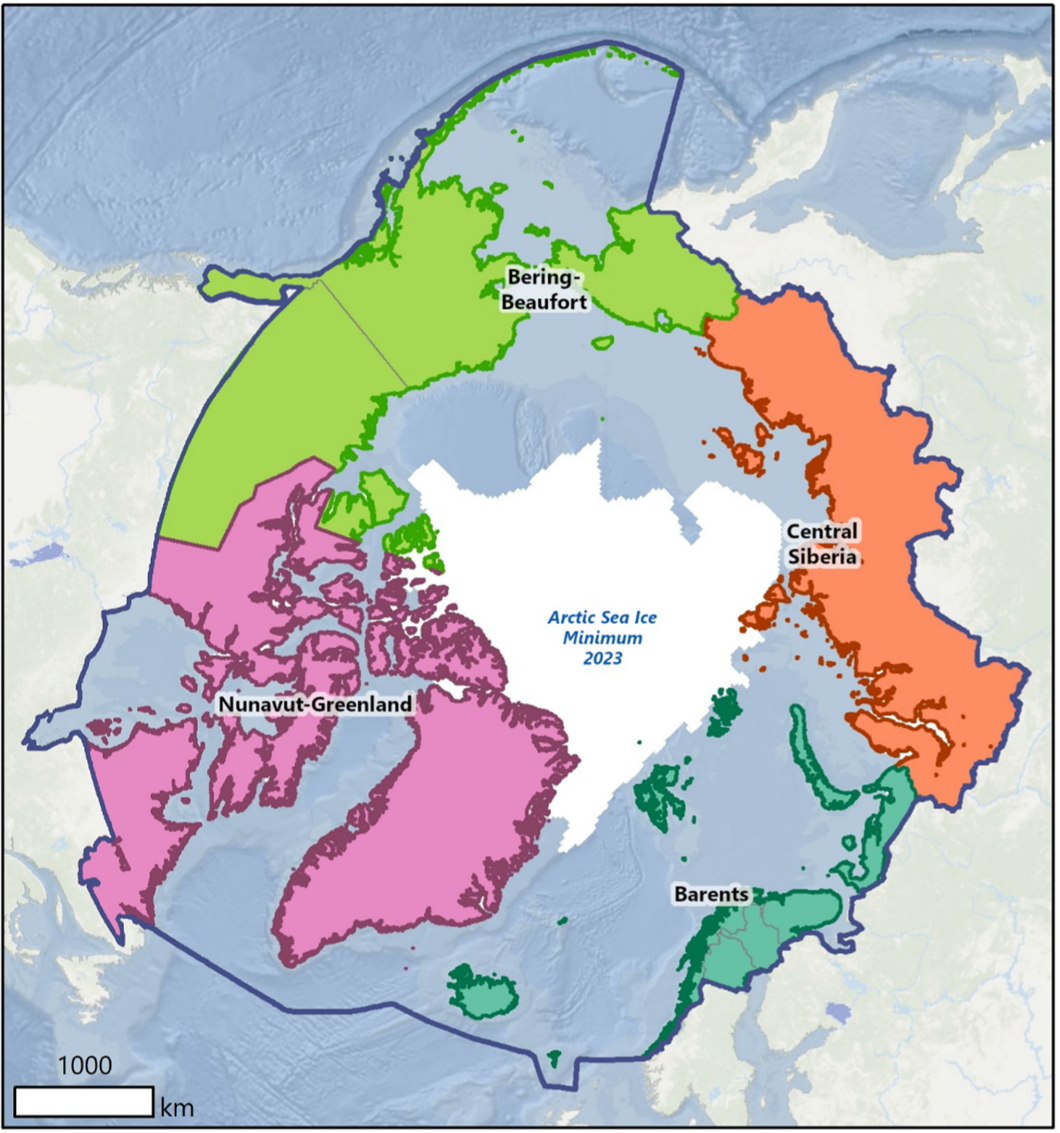
The Arctic divided into four sub-regions

Furthermore, the research team divided the Arctic into four sub-regions to better enable exploration of diversity in potential future outcomes across the Arctic. A map of these four sub-regions—Barents, Bering-Beaufort, Central Siberia, and Nunavut-Greenland—is shown in Fig. 2.

The use of Arctic sub-regions was necessary to enable comparison across the pan-Arctic while limiting the number of questions on the Delphi questionnaires. The research team acknowledges that there is more than one way to subdivide the Arctic and that running an analysis with a different subdivision could produce different results.

The subdivision shown in Fig. 1 focused primarily on cultural and economic factors, even though political and physical geography were also initially considered, because prior experience suggested that development outcomes in any given location in the Arctic may not be completely tied to its national government or national borders. For example, in the Barents sub-region there is a rich history of international cooperation among the countries and Indigenous Peoples. This sub-region spans very different governance structures and political ideologies; cooperation has been facilitated by common goals rooted in shared cultural and economic principles. After the 2022 full-scale invasion of Ukraine, many areas of Barents sub-region cooperation across borders with Russia rooted in economic goals and cultural histories slowed or halted, highlighting that conceptions of similarities and linkages within the Arctic are not necessarily enduring. The discussion section returns to this issue in more detail.

### Identification and prioritization of selected Arctic 2050 outcomes

The Delphi exercise relied on questionnaires about possible Arctic development outcomes in 2050. The research team defined *development* by combining ideas in several sources to arrive at *the act of altering existing infrastructure (fixed or mobile) or land, ocean, atmosphere, and/or electromagnetic spectrum use for the purpose of economic growth or to improve quality of life; or the application of technology for the production of new goods and services* (Arctic Council undated; Arctic Economic Council undated; Wynne Houck 2022). The research team used a dictionary definition for *outcome*, namely *something that follows as a result or consequence* (Merriam Webster undated).

Before designing the Delphi exercise, the research team systematically identified and then prioritized outcomes for use in the exercise. There is a soft limit to how many questions can be included in a Delphi exercise before participation will likely drop off. The research team decided that ten Arctic 2050 outcomes was the maximum number that could be included in the Delphi exercise without significant loss of participation, as the use of ten outcomes would ultimately translate to forty questions (when applied across the four sub-regions in Fig. 1), a substantial time commitment for participants.

Between 2020 and 2021—a period notably affected by the COVID-19 pandemic and before Russia’s full-scale invasion of Ukraine—the research team conducted a traditional literature review, surveyed Arctic conference topics, and conducted interviews with subject matter experts within the International Reference Group and/or based on suggestions from this group. These background activities were used to develop a theoretical concept for Arctic outcomes related to changes in physical, socio-cultural, political, economic, and/or technological factors, as already illustrated in Fig. 1.

The literature review did not employ the methods of either systemic or scoping reviews (e.g., Munn et al. 2018) and used Google Scholar to identify research articles since 2010, roughly a decade prior to the start of the research. Searches covered the five varieties of change factors noted above and used the terms *Arctic*, *far North*, *high North*, and *north* (when associated with the name of one of the Arctic states) to scope to the geography of interest. For example, searches were undertaken on “Arctic + politics,” “far North + economy,” and “Russian north + technology.” Research abstracts and keywords were scanned to identify focus areas and each individual literature search was paused when new topics ceased to be identified. Conference presentations at the 2020 Arctic Circle Assembly and Arctic Frontiers venues as well as interviews were similarly focused on the five varieties of change factors and used for the purpose of educating the research team prior to the Delphi analysis, rather than being formal analytic approaches unto themselves.

The team then engaged in two successive “red teaming” workshops leveraging the diverse expertise of twelve experts (not already interviewed) in Arctic security, demographics, and physical geography to test that the factors and types of outcomes identified covered an analytically and policy-relevant range of possibilities. Red teaming is *an approach within planning or analytic gaming that focuses on challenging assumptions*, a description derived from Sandia National Laboratories (undated).

Next, the research team used the theory of driving factors illustrated in Fig. 1 to feed into a prioritization of ten plausible Arctic 2050 outcome types that would be used in the Delphi exercise (which was constrained in the number of outcomes that could be accommodated) employing the following considerations:

- Likely to have a strong impact on shaping the Arctic’s future but uncertain in direction based on expert insights in literature and interviews, and at conferences. (For example, trends in a particular economic sector are subject to numerous uncertainties and thus worthy of polling expert opinion whereas there is an overwhelming amount of scientific evidence for how climate change will alter the physical geography of the Arctic.)
- Not likely to be uniform across the pan-Arctic based on an understanding of early 2020s social, political, and economic regional geography.
- Diverse in terms of economic sectors and social aspects considered.

The ten selected outcomes (Table 1) fall roughly into two umbrella categories covering selected economic sectors and issues of sovereignty and governance that can drive change in the Arctic. These are neither exhaustive nor mutually exclusive.

**Table 1:** Development Outcomes Included in Delphi Exercise

Outcome	Description of Outcome	Type: Economic or Sovereignty/Governance
Food	Growth in ocean and land-based production	Economic
Oil and natural gas	Accelerated hydrocarbon extraction	Economic
Mining	Expansion of mining options	Economic
Tourism	Rise in demand for eco- and other tourism	Economic
Technology	Expansion of technology sector	Economic
Arctic residency	Increased public desire to establish or maintain residency in the Arctic	Sovereignty/Governance
Indigenous self-determination	Increased collaboration between Indigenous populations and state/federal and/or private companies	Sovereignty/Governance
Multi-stakeholder cooperation	Decline in cooperation over development issues (growing control over decision making at the national level)	Sovereignty/Governance
Protected areas	Reduced access to land and coastal/maritime areas associated with climate change mitigation (e.g., expansion of renewable energy production; decarbonization; ecological conservation)	Sovereignty/Governance
Military operations	Expansion and persistence of military operations	Sovereignty/Governance

Although food production is also important outside of its status as an economic sector, the research team specifically wished to probe the development of aquaculture and agriculture given greater uncertainty with food as an industry as opposed to the enduring importance of food produced in or near communities, which was also a topic included within some of the sovereignty and governance issues.

Oil and gas extraction and mining were included because of their existing status as major industries in the Arctic. Their future, however, particularly outside of Russia, is uncertain given public and governmental pressures to act on climate change with mitigation and adaptation strategies and move toward renewables. Hydrocarbons and mining were considered separately given that while the former exacerbate climate change, the latter may mitigate its effects by supporting a green energy transition.

Both tourism and technology are key economic sectors today in different parts of the Arctic. Visits to Iceland, Finland, and Norway have been growing rapidly for years, while southeast Alaska has strong cruise tourism. Cruise tourism is also growing in Norway, both along the coast and around Svalbard, and in Greenland. In contrast, the annual cruise from Murmansk, Russia to the North Pole aboard a nuclear icebreaker, *50 Years of Victory,* which was operated by UK-based Poseidon Expeditions, has been suspended since 2022, when Russia invaded Ukraine. With regard to telecommunications developments, the Nordic countries have been leaders in this sector and in other high-tech industries, like data centers, for years (Sovacool et al. 2022), whereas many other parts of the Arctic are not typically known for “high tech.” The unevenness of these industries across the Arctic made it interesting to examine whether this could change over the coming decades.

Many parts of the Arctic are currently demographically static or experiencing population decline. Governments such as those in Norway, Canada, and Sweden have tried to counter this trend by directing immigrants and refugees to cities like Tromsø, Yellowknife, and Kiruna (e.g., Brouwer 2019). A desire to stay in or move to the Arctic depends on factors such as food and water security as well as availability of livelihoods and services. Kiruna, for instance, often attracts people from southern Sweden and elsewhere across the European Union interested in the outdoors, yet anecdotally a lack of sports doctors is sometimes mentioned as a challenge to staying in the Arctic long-term (Author’s field notes, 2022).

Indigenous self-determination is a key governance factor that varies considerably across the Arctic (Nicol 2010). The degree to which Indigenous institutions make decisions at various scales on behalf of their communities is an important factor in whether and how cash economic ventures shape the future as well as the types of social initiatives prioritized including education in traditional languages. Between the time we completed our Delphi exercise and the publishing of this paper, Canada reached a devolution agreement with Nunavut in 2024 (Government of Canada 2024; Crown-Indigenous Relations and Northern Affairs Canada 2024); Alaska formally recognized Alaska Native Tribal authority in 2022 (Samuels 2022); Greenland released a draft constitution in 2023 and remains in ongoing talks with Denmark about independence (Wehmeyer 2023); and in Scandinavia the Sámi people won both human and constitutional rights cases before the Norwegian (2021) and Finnish (2022) Supreme Courts (Library of Congress Finland 2022; Reuters 2023).

For decades, Arctic dialogue and cooperation among states as well as rights-holders and other actors has been relatively or highly multi-lateral. Historical cooperation has roots, for instance, in Arctic Indigenous Peoples’ organizations. The Sámiráđđi (Sámi Council) was founded in 1956 and the Inuit Circumpolar Council was founded in 1977, both pre-dating the Arctic Council, established in 1996 by the Ottawa Declaration, by decades.

However, Russia’s annexation of Crimea in 2014 and full-scale invasion of Ukraine in 2022 initiated a split in cooperation within several Arctic institutions. For example, Russia disengaged from the Barents-Euro Arctic Council in 2023 and suspended funding to the Arctic Council in 2024, even though it remains a member along with the seven other Arctic countries—all of which are now NATO members. At the same time, the Central Arctic Ocean Fisheries Agreement’s Committee of the Parties convened for the first time in 2022, including Russia in its meeting (Koivurova and Shibata 2023), demonstrating the resilience of Arctic multilateralism within narrower sectors.

Growth of protected areas and military activity are very different types of outcomes which can control mobility and other forms of activity. Areas can be protected for biodiversity, to ensure access to traditional livelihoods and cultural practices, as parks, and/or to support climate change mitigation (e.g., a carbon sink). Military areas tend to be restricted from public use and may also have barriers that also inhibit wildlife movement. Military exercises, tests, and other activities may incur temporary or long-term hazards that also restrict movement in and around these areas, development of communities, and use of space for commercial purposes. Yet in other parts of the world, the creation of no-go zones and demilitarized zones as in Chernobyl and between North and South Korea has led wildlife to flourish (e.g., Deryabina et al. 2015; Brady 2021).

### Delphi 2050 forecasting exercise

The Delphi method (Helmer-Hirschberg 1967; Stone et al. 2005) has been widely recognized as a structured expert elicitation tool since it was pioneered in the 1950s to forecast the impacts of technology on warfare. It has since been employed more broadly to technology forecasting and to research on education and healthcare outcomes. The Delphi approach has been used to explore a variety of topics in northern locations such as Finnish maritime technology (Myllylä and Kaivo-oja 2015), the Icelandic economy (Steindórsdóttir 2017), cruise tourism in Canada (Dawson et al. 2016), and suicide prevention (Collins et al. 2019).

**Fig. 3:**
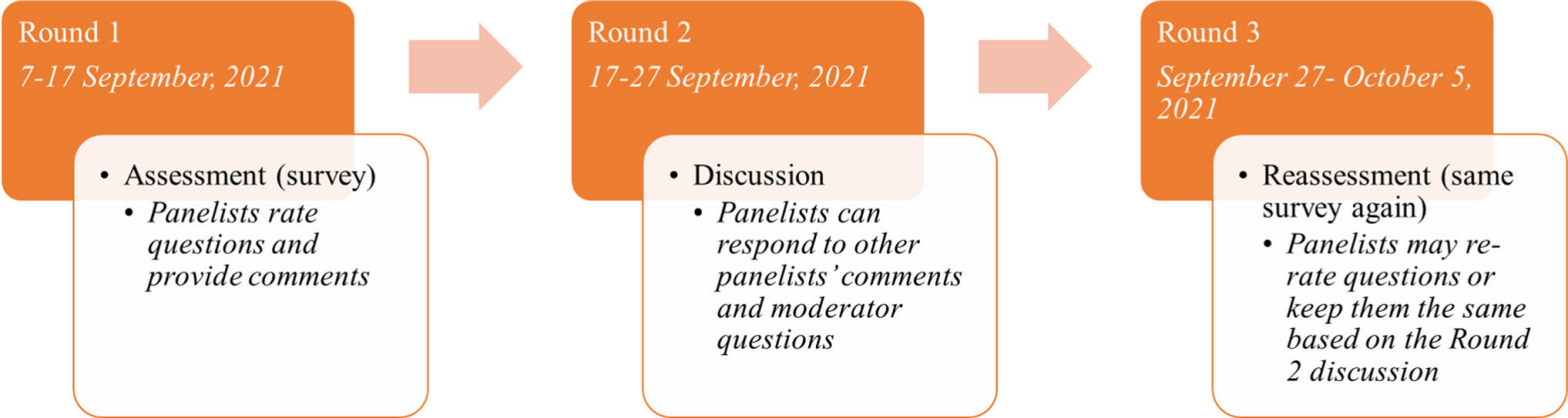
Summary of Delphi method timeline

This Delphi exercise was conducted anonymously using the ExpertLens software (Dalal et al. 2011) over three rounds held in September 2021, as summarized in Fig. 3. The first and third rounds involved a detailed questionnaire asking about the qualitative likelihood of the outcomes in Table 1 occurring in each of the sub-regions by 2050. Outcomes were translated for the Delphi exercise into questions in the form of: *How likely is it that the X region will experience Y outcome by 2050?* Participants responded to questions using a five-point Likert scale, with one representing “very unlikely,” three indicating “just as likely as not,” and five meaning “very likely.” During the second round, first round results were revealed in aggregate and participants were able to discuss them online within the ExpertLens platform.

ExpertLens also captured any freeform written comments participants chose to offer alongside their ratings. These were primarily used by the research team to seek additional context for patterns in the questionnaire ratings. The research team also selected a subset of terms relevant to the Arctic outcomes and conducted a frequency analysis to ascertain some topics of particular emphasis within the written comments.

Experts were recruited through professional connections and via the website and social media of Arctic Frontiers, an organization based in Tromsø, Norway, which has hosted a major conference on the Arctic each year since 2006 (Arctic Frontiers undated, Steinveg 2021). Recruitment was also done within academia, industry, government, and Indigenous Peoples’ organizations. Ultimately, 78 participants agreed to receive an emailed invitation to participate, of which 56 participated in the first questionnaire, 29 engaged in some form of dialogue during the second round, and 34 participated in the second questionnaire. The number of participants who answered almost all the questions was somewhat less – 40 for the first questionnaire and 26 in the second. All data were used, regardless of how completely the participant participated.

## Results

**Fig. 4:**
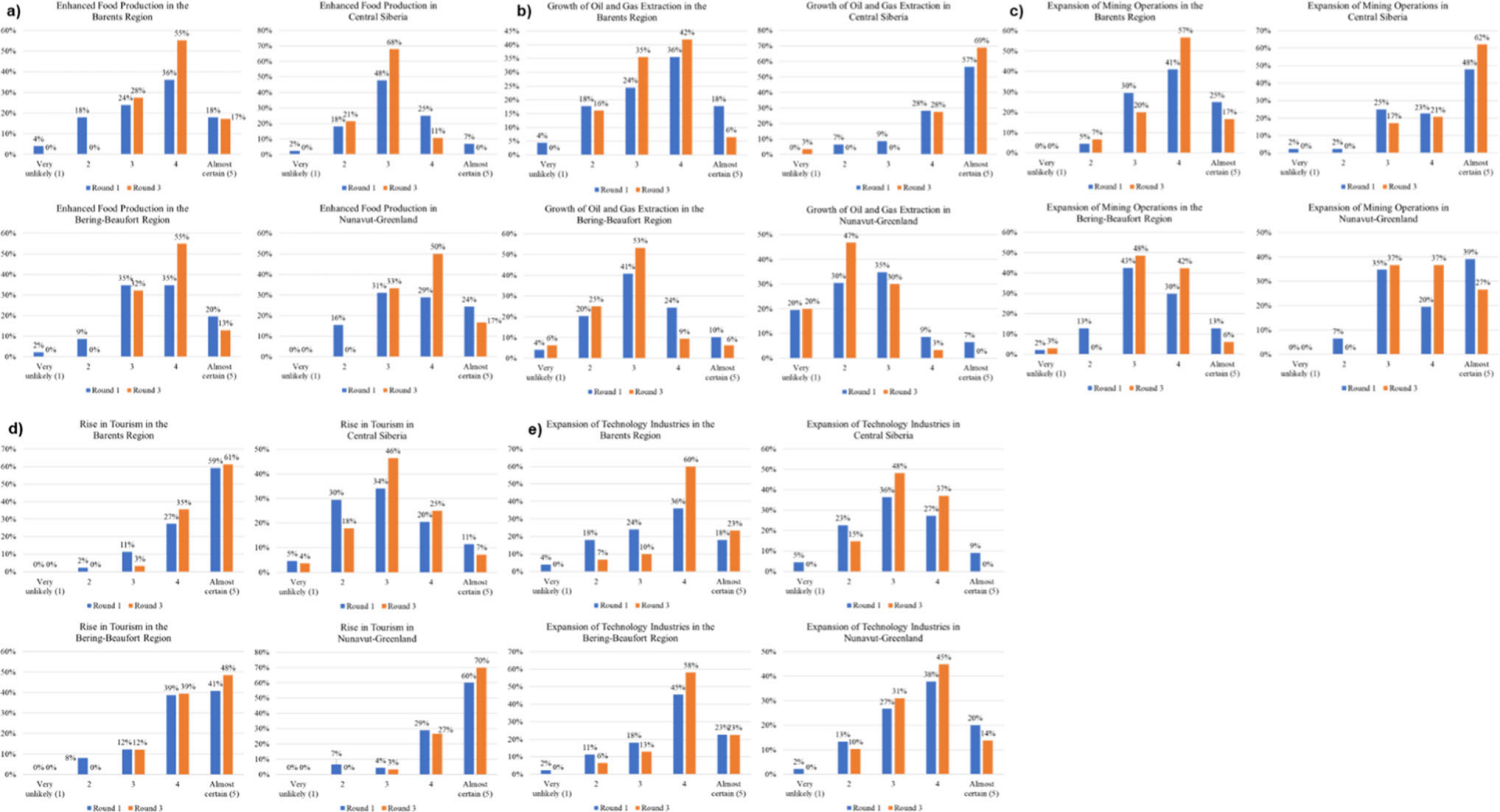
Summary of Delphi results for economic sectors

**Fig. 5:**
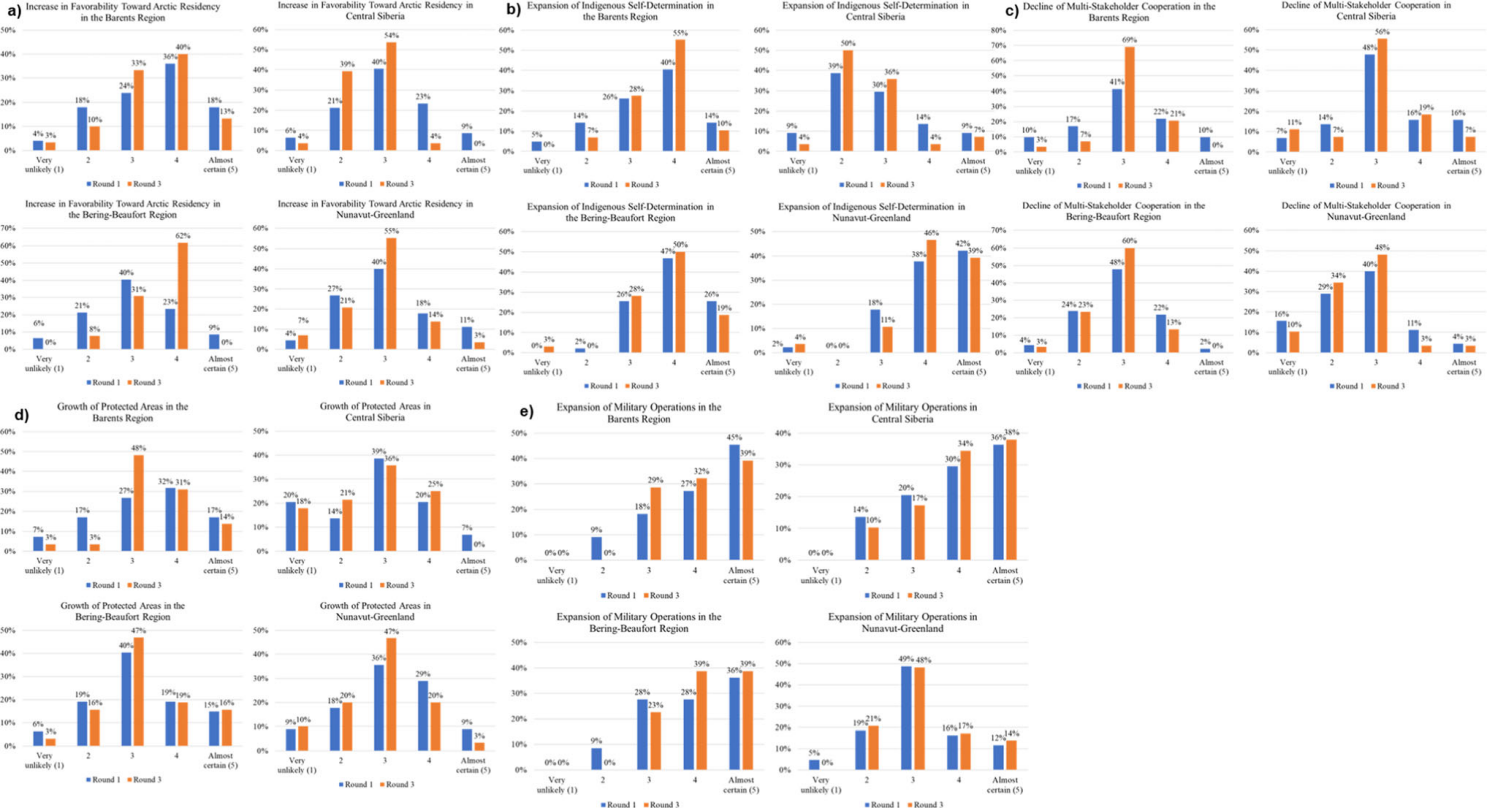
Summary of Delphi results for sovereignty and governance issues

The quantitative results for each outcome are included in Figs. 4a-e and 5a-e. Each graph demonstrates participant responses across the first and last rounds (the ones that included the questionnaire) for each of the four sub-regions. Following these quantitative Delphi results are supplemental qualitative findings summarizing the quantitative results and (separately) based on the written comments of participants, which provide important additional context for interpreting the results in Figs. 4 and 5. Larger versions of the graphs in Figs. 4 and 5 can be found in the Supplementary Information.

Participants built some consensus around food production outcomes (Fig. 4a) in different parts of the Arctic and were overall relatively optimistic about growth in food production in the Barents, Bering-Beaufort, and Nunavut-Greenland sub-regions. Majority scores of 4 (“likely”) across participants in round 3 for these sub-regions primarily reflect shifts away from lower scores (less likely) in round 1 and some movement away from scores of 5 (“almost certain”) following discussion between rounds. Of these three sub-regions where food production was overall expected to grow by 2050, there was most disagreement for Nunavut-Greenland; although half of round 3 responses indicated “likely” there were also a substantial number of participants that rated this outcome a 3 (“just as likely to happen as not”) and a 5 (“almost certain”).

The exception to anticipated growth in food production is Central Siberia. Most participants rated this “just as likely to happen as not” in round 1, an assessment which gained further agreement by round 3.

Results for growth of oil and gas extraction (Fig. 4b) were almost mirror opposites to those for food production above. In this case, Central Siberia was the only sub-region where almost all participants suggested growth. The spread of responses across the other sub-regions was less consistent, with the only majority agreement reached by round 3 being that an increase in extraction would be “just as likely to happen as not” for the Bering-Beaufort sub-region. Most participants thought extraction could increase in the Barents and many estimated that extraction was unlikely to increase in Nunavut-Greenland, the latter of which is also consistent with little or no offshore oil and gas being found in the sub-region and/or these resources being extremely expensive to extract.

Results suggest strong expectations for mining (Fig. 4c) to grow in Central Siberia and a good likelihood of this outcome in the Barents sub-region. Participants built far less consensus for the Bering-Beaufort and Nunavut-Greenland sub-regions, though there was some shift between rounds 1 and 3 away from extremes. For each of these two sub-regions there were a substantial number of 3 ratings (“just as likely to happen as not”) in both Delphi questionnaires, as well as a shift in 2 ratings (“unlikely”) in round 1 to ratings of 3 (“just as likely to happen as not”) and 4 (“likely”) in round 2. The number of participants offering ratings of 5 (“almost certain”) in round 1 decreased by round 3 for both sub-regions as well, especially for Nunavut-Greenland.

Most participants strongly expected tourism to rise (ratings of 4 or 5 in Fig. 4d) for all sub-regions except Central Siberia. Central Siberia saw a considerable spread of expectations for tourism with almost half of participants suggesting a rise in tourism was “just as likely to happen as not” by round 3. Ratings of 5 (“almost certain”) increased from round 1 to round 3 for all the sub-regions except Central Siberia. This may follow historically low patterns of international tourism to Russia, which do not appear poised to increase, at least from western countries, in light of limited diplomatic and economic relations. Any optimism reflected in tourism to Central Siberia may reflect expectations that this could increase via domestic populations. Central Siberia also lacks appropriate infrastructure in many locations to support growing tourism and the overwhelming focus on resource extraction is likely at odds with growing a strong tourism industry in this area.

Many participants felt that there would be expansion of technology industries (Fig. 4e), especially in the Barents and Bering-Beaufort sub-regions. Most participants rated this outcome a 4 (“likely”) for the Barents and Bering-Beaufort sub-regions by round 3, and almost a fourth rated this outcome a 5 (“almost certain”) for these sub-regions by round 3. There is a similar pattern for Nunavut-Greenland, though with slightly more pessimism than for the two previously discussed sub-regions, as reflected in a slightly higher number of 2 ratings (“unlikely”) and 3 ratings (“just as likely to happen as not”). Central Siberia has a broader spread of expectations, with almost half of participants suggesting technology sector expansion as “just as likely to happen as not” by round 3. Central Siberia also saw slight declines in 2 ratings (“unlikely”) and 5 ratings (“almost certain”) between rounds 1 and 3.

Participant ratings for increases in Arctic population (or those residing there) (Fig. 5a) were spread across 2 (“unlikely”), 3 (“just as likely to happen as not”), and 4 (“likely”) for all sub-regions. By round 3, however, there was a strong increase in optimism that living in the Bering-Beaufort sub-region of the Arctic would be seen as “likely” to be favorable by 2050. There was a smaller jump in “likely” ratings for the Barents sub-region by round 3, which also had many responses of “just as likely as not” by round 3. The rating of 3 (“just as likely as not”) rose to a slight majority of participants for Central Siberia and Nunavut-Greenland by round 3.

A vast majority of participants rated an expansion of Indigenous self-determination (Fig. 5b) in Nunavut-Greenland as “likely” or “almost certain” by 2050 in both rounds 1 and 3. A vast majority of participants were relatively pessimistic about this outcome in both rounds for Central Siberia; the vast majority rated this outcome as “unlikely” or “just as likely as not.” By round 3, around half of participants indicated that an expansion of Indigenous self-determination policies was “likely” by 2050 both in the Barents and Bering-Beaufort sub-regions.

Most or many participants selected “just as likely as not” as the outcome for a decline in multi-stakeholder cooperation (Fig. 5c) by 2050 for all sub-regions. Participants appear to have been slightly more optimistic about the possibility of cooperation across stakeholders in the Bering-Beaufort and Nunavut-Greenland sub-regions as seen in the slightly higher number of participants using 2 ratings (“unlikely” to see a decline in cooperation).

Participants expressed disagreement on whether protected areas would grow (Fig. 5d) in the different sub-regions. By round 3, the largest fraction of responses fell into the “just as likely as not” category. The number of 5 ratings (“almost certain”) was highest in the Barents and Bering-Beaufort sub-regions. The number of 1 ratings (“very unlikely”) was highest for Central Siberia, followed by Nunavut-Greenland.

A vast majority of participants felt that military operations would expand (“likely” or “almost certain” in Fig. 5e) in the Barents, Central Siberia, and Bering-Beaufort sub-regions. Almost half of participants thought that military operations expansion in Nunavut-Greenland was “just as likely as not.”

Table 2 summarizes outcomes by sub-region based on the one, two, or three most common round 3 ratings by participants. Only one rating is provided if at least 50% of participants selected this in round 3 for that sub-region and outcome. If no rating was selected by 50% or more of participants in round 3, then the first and second-most selected rating is represented in the table. In rare cases, the second- and third-most selected ratings were so close in range (0–3 percentage points) that a range of values is represented in the table. Note that for convenience, the outcome on cooperation for Nunavut-Greenland is reversed (i.e., represents the likelihood of an increase—as opposed to decrease—in cooperation).

**Table 2:**
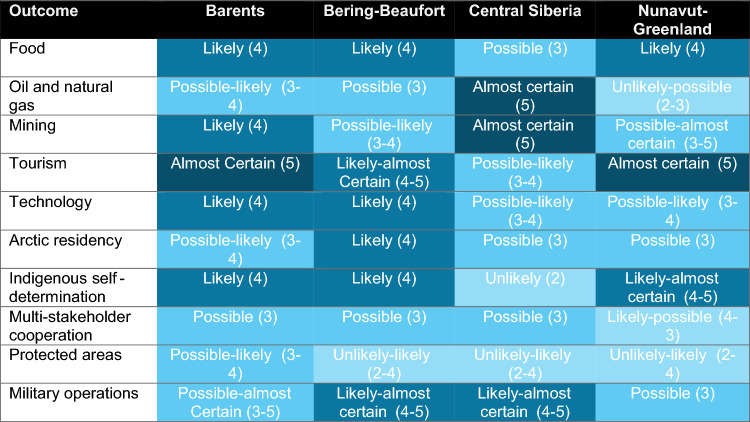
Summary of outcomes by sub-region

Colors in the table correspond to the lowest rating provided

In this table, the most consistently likely outcomes include food production, tourism, technology, and military operations. The most consistently unclear (“just as likely as not” ratings) across sub-regions relates to protected areas. Variability in expert expectations across sub-regions is most pronounced for oil and natural gas production and mining.

Table 3 summarizes participant comments collected during the Delphi process. Although we are unable to present every comment, the analysis captured themes and perspectives that arose repeatedly and are relevant for interpreting the response patterns captured from the Delphi questionnaires.

**Table 3:** Summary of selected themes and insights from Delphi participant written comments across rounds 1 and 3

Barents	• Warming will open more opportunities for land-based food production; ocean food production already high and likely to continue due to demand and fish stocks potentially shifting north
	• Oil and gas projects still being considered by Norway and Russia despite expectations for ultimate decline in global demand
	• Global desire to see the changing Arctic and relatively good transportation/hospitality infrastructure will drive tourism industry
	• Public overall favors mining and global demand for minerals expected to go up
	• High use of coastal and marine areas suggest difficulty in setting aside areas for conservation
	• Given declining or flat populations in this region there may not be a big population increase; given sufficient housing, services, and difficult living situations at lower latitudes some population increase could occur
	• There is ongoing dialogue between Indigenous and state/private companies but the outcome of that is not yet clear
	• Technology (e.g., in space) is expanding in general which will have an effect in the Arctic; the Nordic countries have a relatively strong innovation sector and tech clusters are continuing to appear/grow
	• Russia has been very militarily active for years and military presence overall is already expanding in this region, a seam between Russia and Western European countries
Central Siberia	• Russian government sees Arctic as a region with many resources and that these should be extracted for economic survival
	• A large percentage of Russia’s economy is reliant on oil and gas production and countries like China are interested in increasing importation of these products
	• The region is mineral rich and global demand for commodities is rising
	• Any growth in technology would be related to the resource extraction industry
	• Very few/no opportunities for local perspectives to influence national policies; equity is not prioritized
	• Unappealing for tourism due to focus on extractive industries, limited infrastructure to support visitors, and domestic politics
	• Environmental damage from extractive activities could limit any potential for expanded food production
	• Several areas with non-permanent populations; need investment in infrastructure and long-term livelihoods to encourage population growth
	• Military capabilities focused on coasts and islands, not inland; this region does not have any international borders to manage
Bering-Beaufort	• Strong debates in Canada and U.S. on future of extractive activities engenders uncertainty for the future of oil, gas, and mineral industries; on Russian side much more firm in long-term plans to continue these activities
	• Dialogue with Indigenous and other local communities is important but unevenly done across the region
	• Alaska particularly has a growing tourism industry focused on unique ecology and wilderness areas, and leveraging existing transportation infrastructure
	• Changing climate could increase opportunities for ocean- and land-based food production; will be limited by number of people to work in food-related industries; food security could also be threatened by loss of access to traditional/subsistence food sources
	• Cost of living and constrained access to services limits desirability for living
	• Potential for increased access to technologies such as broadband communications and for resource extraction
	• Other needs (e.g., for transportation and energy) might preclude development of additional protected areas
	• Rising tensions in the region could draw additional military activity; also continues to be strategic location
Nunavut-Greenland	• Sovereignty and Indigenous self-rule are central issues
	• Important ongoing debates on circumstances under which increasing mining activities would benefit local communities
	• Strong Indigenous voices against hydrocarbon extraction and lack of existing infrastructure limits these industries
	• Diversity in ecosystems and culture will help drive tourism
	• Indigenous communities will remain in region; in-migration from outside areas unlikely unless access to varied livelihoods and services improves
	• More capacity already exists for ocean-based food; some potential with warming for land-based production
	• There is no evidence of strong plans to build any technology hubs; space-based communications have potential to increase connectivity
	• Needs for manufacturing, transportation, energy, and waste solutions if population and economy are to expand
	• Relatively high level of human agency and decolonization may encourage a more collaborative environment
	• Canada especially has high targets for conservation
	• Military roles limited primarily to public service (except Pituffik Space Base) instead of hard security, though geopolitical competition could impact in future

**Fig. 6:**
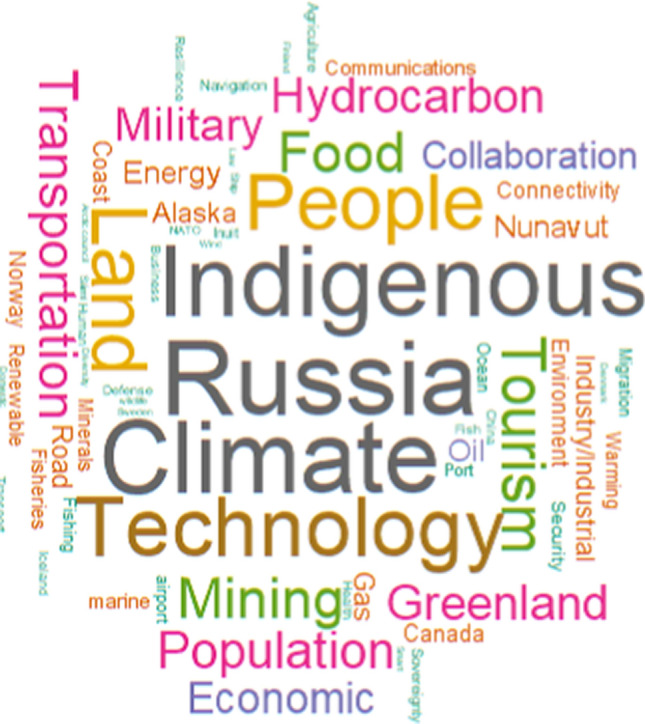
Word cloud depicting the relative frequency of selected words used in comments by Delphi participants in rounds 1 and 3

Figure 6 demonstrates another perspective on Delphi participant insights across rounds 1 and 3. After reviewing comments, the research team selected keywords representing different nations and places, industries, and activities and calculated the relative frequency of their use in the results. The keywords were selected based on their clear regional connection (e.g., the names of the different Arctic countries) and association with the ten outcomes based on a thorough visual inspection of the results from the freeform written responses. The research team did not attempt to exhaustively analyze the full range of keywords in the text, so this depiction has limited perspective, though it covers a range of issues discussed by participants. The words represented in this figure include those with at least three distinct mentions in the written comments across all sub-regions.

## Discussion

Although the Arctic is often discussed as a homogenous whole, the region’s physical heterogeneity has long been documented and increasingly diversity along socio-cultural, demographic, economic, and political dimensions has also been captured, for instance, by the AHDR volumes, AMSA report, Heinämäki et al. (2014), Fondahl et al. (2015), and Middleton et al. (2019, 2021). Building on this literature, our study of potential future economic sectors and sovereignty/governance outcomes across the Arctic demonstrates that continuing differences may be persistent and that diverging socio-economic trends are not just plausible but should also be expected. These findings lend additional analytic credence to the concept of “many Arctics” (Łuszczuk 2017; Dodds and Smith 2022; Spence et al. 2023). This discussion will first deliberate on the research findings related to results about the four Arctic sub-regions and then consider how impacts from the ongoing war in Ukraine at the time this manuscript was prepared introduce a new geography to the “many Arctics” concept.

Across all four sub-regions, there appear to be the most similarities in expected outcomes by 2050 between the Barents and Bering-Beaufort sub-regions. This is surprising in some ways, given present differences in physical geography and climate, demographics, cultures, infrastructure, economies, and modes of subsistence and mobility. This being said, perceived similarities in trajectories may reflect shared areas of potential opportunities and challenges in the future, and current overlap in some values between the Nordic countries and North America (though both of these sub-regions also include parts of Russia). There are also some key differences in anticipated outcomes between these sub-regions, and among all sub-regions, as discussed below.

In the Barents sub-region, most outcomes were deemed by experts to be fairly likely, potentially reflecting the relatively high inhabitation, infrastructure, investment, and civil dialogue. It was also recognized as a potential zone for geopolitical tensions that could work against economic development and the wellbeing of Indigenous and other communities.

There was uncertainty expressed about extractive activities in the Bering-Beaufort sub-region, reflecting shifting environmental policy and continuing discussions on regulations, Indigenous and other local community participation options, and environmental and cultural protection. Strong potential for growing ecotourism as well as military operations were projected as well. Both make sense in the present-day context in which Alaska, for example, is home to many strategic U.S. military assets and booming cruise tourism, which is under pressure to become more sustainable. Russia also holds strategic military assets in the sub-region, and the Bering Strait waterway provides important access to the Northern Sea Route (NSR), which Russia sees as very important to its future (e.g., Mikhailova and Tabata 2024).

In contrast, there was high certainty that extractive activities would continue for decades in Central Siberia, consistent with the importance the Russian government places on developing natural resources in its north. In 2018, 17% of Russia’s crude oil production came from the Arctic, but the government aims to boost that proportion to 23% by 2030 (Gontmakher 2022). The rich hydrocarbon and mineral reserves of this region combined with low population density and environmental stakeholders’ limited abilities to influence governance add some logic to this projected set of outcomes. Indeed, experts were most pessimistic about opportunities for cooperation between Indigenous communities and the (Russian federal) government in this sub-region. Given a lack of connections to the West, it is possible that Central Siberia may diverge even further in terms of its various trajectories from the other three sub-regions. This could make the knowledge of Indigenous scholars, such as that highlighted in Ksenofontov and Petrov (2024), ever more important for communicating information about this sub-region to the outside.

Finally, Nunavut-Greenland showed much potential for leading the way in Indigenous self-determination. This is unsurprising given the prominence of Indigenous leadership in decision making in these areas at present (Grydehøj 2020). Greenland has been working toward a more direct relationship with the United States as seen, for example, through recent years’ negotiations regarding Pituffik Space Base (formerly Thule Airbase) (Olsvig 2024), and Nunavut continues to gain governance decision-making authorities, notably as with the 2024 Devolution Agreement referenced earlier, which was signed after the Delphi research. Tourism was projected to rise, though this implies additional infrastructure needs to support expanded capacity for visitors. For example, runways at airports in Nuuk and Ilulissat are being extended to allow larger, longer-haul aircraft to safely land (e.g., Casey 2023). The diversity of responses with respect to the potential for future mining certainly reflects ongoing debates in Greenland regarding whether and how to translate potential mineral wealth into benefits for Greenlandic society.

This work illustrates but one way of representing heterogeneity across the Arctic and the “many Arctics” concept. The worsened split between Russia and the other seven Arctic states following its full-scale invasion of Ukraine also lends geopolitical credence to the emergence of more than one Arctic—in this case, a highly divided, bipolar region. The war has compelled Finland and Sweden to join NATO, meaning that now, seven out of eight Arctic states—all but Russia—are members of that alliance. Weakened circumpolar cooperation portends worse outcomes for Indigenous Peoples, the environment, public health, pollution, and the many other groups and processes that span international borders. A breakdown in relations also will make it harder to study these disparities in person. While technologies such as satellite remote sensing may make it feasible for Russia and the other Arctic states to observe one another from a distance, the people-to-people connections that have driven knowledge sharing across the region since the end of the Cold War are now in serious jeopardy. Even digital connections offer scant assistance in overcoming these hurdles given fear among many Russians of the potential repercussions of interacting with foreigners, and the restrictions placed on researchers and practitioners in other Arctic states on collaborating with Russians.

The bipolar splitting of the Arctic described above could continue to dominate the way the “many Arctics” concept plays out in practice at least until the geopolitical situation in the Arctic shifts again. Yet inherent in the “many Arctics” concept is the idea that there can be different realities at the same time. The logic behind the four sub-regions identified for the purposes of this research was primarily grounded in geographic proximity, cultural ties and history, and economic linkages. Despite current geopolitical tensions, those other linkages still exist even if some connections are less accessible or paused as of 2024. For example, Sámi and Inuit communities are still tied by family connections and traditions, even if in-person engagement across borders with Russia isn’t feasible. The U.S. and Russia continue to manage their shared border in the Bering Strait, and Norway and Russia continue to engage on the management of their shared fishery in the Barents Sea.

Having “many Arctics”—whether due to socio-economic differences or geopolitical tensions or both—makes finding common priorities for terrestrial and marine use and management increasingly difficult, which will influence the types of collaboration and governance approaches that persist or form. It is possible that further tensions could arise over resource extraction and environmental protection due to differences in activities and plans across the Arctic. For example, fish are a mobile resource; differences in stock management and rules enforcement across maritime lines can lead to uneven sharing of benefits and costs. As fish species move farther north in pursuit of colder waters, this could also upend traditional fishing grounds and put pressure on the current agreement by the five Arctic coastal states, the European Union, China, Japan, and South Korea to refrain from fishing in the Central Arctic Ocean (e.g., Dyck 2024).

With both climate change and geopolitical tensions challenging strong coordination across the Arctic, opportunities to facilitate more spatially even outcomes, whether through equitable economic development or ensuring food security across Arctic communities, could also be missed. For example, knowledge sharing, financing, workforce development, and logistics coordination among countries can help produce more economically viable, equitable, and environmentally responsible mining operations. In the absence of such exchange, existing environmental disparities across the Arctic could be exacerbated. For example, Russia may double down on fossil fuel extraction to generate revenues for an economy cut off from the West, worsening the impacts of both pollution and climate change for people living close to these deposits. A renewed focus on militarization across the Arctic could also draw away attention from other issues like public health and the environment, from Alaska to Norway. More research on the relationships between regional socio-economic and biophysical variables and how they vary across the Arctic is necessary. This could be guided by frameworks such as the shared socio-economic pathways (SSPs), which identify five potential trajectories for coupled human development and global environmental change in the twenty-first century (Van Vuuren et al 2017).

Finally, while some parts of the Arctic are generating more observations and data than ever thanks to the proliferation and integration of technologies like satellite data, social media, and mobile phones, others are getting left behind due to the digital divide. Even prior to the 2022 full-scale invasion of Ukraine, ties between Russian and Western cyberspace and knowledge communities were dwindling. Post-2022 there have also been intense disruptions to Arctic science, communication between communities, and other forms of knowledge sharing. Determining ways to ensure that years of necessary and beneficial circumpolar collaboration are not completely lost will continue to be challenging.

This research has several shortcomings, which must be noted. Although Delphi participation was reasonably consistent, the research would have benefited from additional participation and perspectives. In particular, it was difficult to communicate with experts within Russia even prior to the full-scale invasion of Ukraine in 2022. The research was also limited by the number of outcomes (10) that were feasible to include in the Delphi, though even this number may have been pushing the limits of expected participation given how many participants dropped out after the first round or did not even start the exercise. Based on feedback from participants, the Delphi also challenged the limits of any given expert’s knowledge because it covered so many topics and different areas of the Arctic. Thus, the results may in part reflect assumptions and/or strong influence from certain participants particularly knowledgeable about certain regions and willing to participate in the Round 2 discussions.

The analysis of the written Delphi comments relied primarily on subject matter expertise to identify themes and insights and did not attempt a more structured qualitative approach such as coding or in-depth analysis of keyword frequencies. This less structured approach was intentional after initial structured analysis revealed inconsistent depth in participant responses. The Delphi instructions may have also contributed to the high frequency use of certain words (e.g., transportation) that may have skewed the results of any frequency analysis, so the research team elected to not conduct such an analysis. Future Delphi research could also evolve the methodology to better incorporate Indigenous knowledge alongside observations and foresight by scholars and the business community. Future research could also consider focusing on one sub-region at a time to allow for more targeted recruitment of regional expertise and richer discussion of local factors that might influence variability in outcomes at this scale.

## Conclusion

Climate change is having widespread impacts across the Arctic. There is sub-regional and local variability not only in how these geophysical impacts are felt and realized, but also in how economic, political, social, and technological factors are shaping ongoing and future changes in uncertain ways. This research illustrates through systematic inquiry one realization of different trajectories in various parts of the Arctic. In so doing, it lends additional evidence toward the “many Arctics” concept and highlights the complexity of driving factors and uncertainties influencing the future of the Arctic, especially economic development and associated governance and decision making.

The fact that diverging trajectories across the Arctic are not just plausible but should also be expected has implications for Arctic development and governance. This became poignantly evident in the aftermath of Russia’s full-scale invasion of Ukraine in 2022, just after the Delphi exercise was completed, which has since witnessed a bipolar splitting of the Arctic along geopolitical fractures, disrupting local to pan-Arctic cooperation in several instances. Yet despite these tensions, the differences and similarities in anticipated future outcomes across the four sub-regions defined for this study remain plausible. Many dimensions of life and livelihoods in the Arctic have not slowed to a halt, although geopolitics is front-of-mind for many policymakers and certain communities and economic sectors are feeling the resulting painful rift. In other words, it appears that the Arctic is experiencing at least two “many Arctics” realities in 2024—the type of socio-economic divergence described across the four sub-regions defined for this study and a split due to a geopolitical rift that became intensively polarizing in 2022. Thus, an emerging challenge for pan-Arctic governance is managing change under circumstances where there are different, co-existing splits across the Arctic that do not resonate. In turn, there are a growing number of obstacles to continuing to build a shared pan-Arctic pool of knowledge and priorities. Implementing Arctic-wide solutions that recognize the region’s many differences while trying to overcome deep and persistent social and environmental inequities and geopolitical tensions will be even harder. Research such as that presented here can help to articulate these issues and some of the key differences across the pan-Arctic, which can both bolster the importance of pan-Arctic governance organizations such as the Arctic Council, as well as support the creation of collaborative policy and research agendas that recognize the Arctic’s heterogeneity across many dimensions.

## Supplementary Information

Below is the link to the electronic supplementary material.

Supplementary file1 (PDF 657 kb)

## Footnotes

1 The International Reference Group is composed of 13 individuals who were recruited by the research team members on the basis of their expertise in areas such as Arctic shipping, Indigenous affairs, geopolitics, and economic development.
