# Supplementary material for: Divergent trajectories of Arctic change: Implications for future socio-economic patterns
Source: Ambio. 2024 Oct 30;54(2):239–55. doi: 10.1007/s13280-024-02080-x (PMC11662104; doi:10.1007/s13280-024-02080-x)
Supplement: Supplementary file 1 — Supplementary file1 (PDF 657 kb) [file 13280_2024_2080_MOESM1_ESM.pdf]

Ambio

Supplementary Information

This supplementary information has not been peer reviewed.

Title: **Divergent trajectories of Arctic change: Implications for future socio-economic patterns**

This supplementary information contains larger versions of individual graphs within Figures 4 and 5 as well as additional information regarding the conduct of the Delphi exercise and the Delphi exercise protocol in its entirety.

Larger versions of individual graphs within Figures 4 and 5

Figure 4a. Delphi results for food production

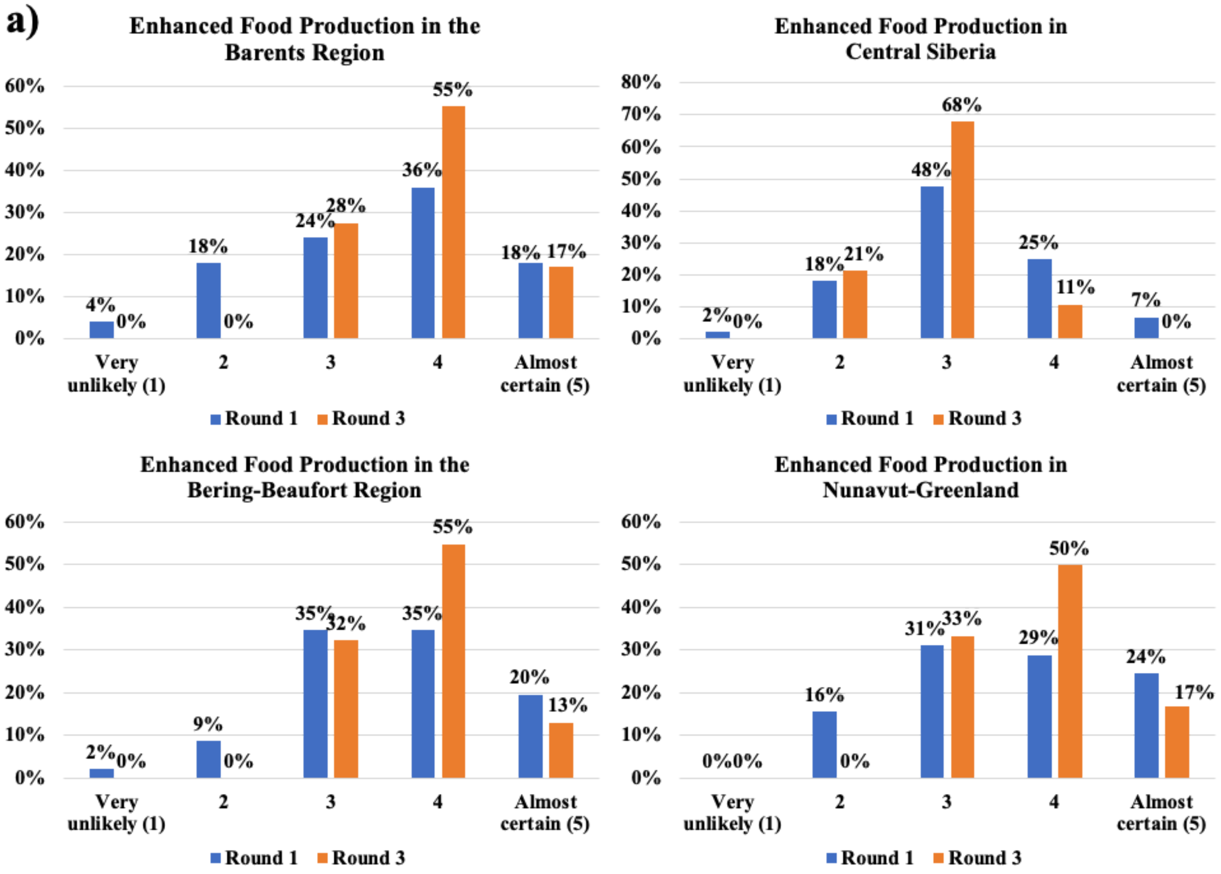

Figure 4b. Delphi results for oil and gas extraction

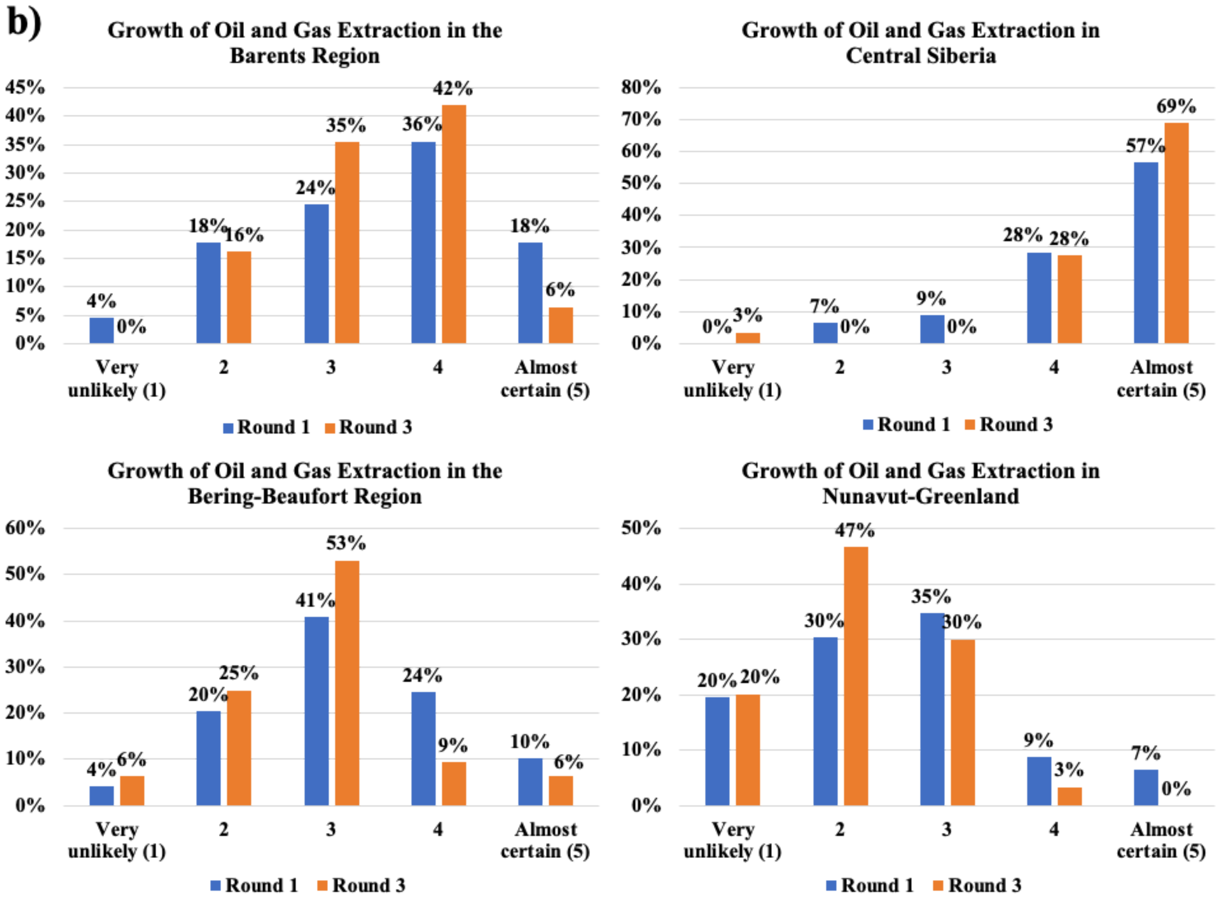

Figure 4c. Delphi results for expansion of mining

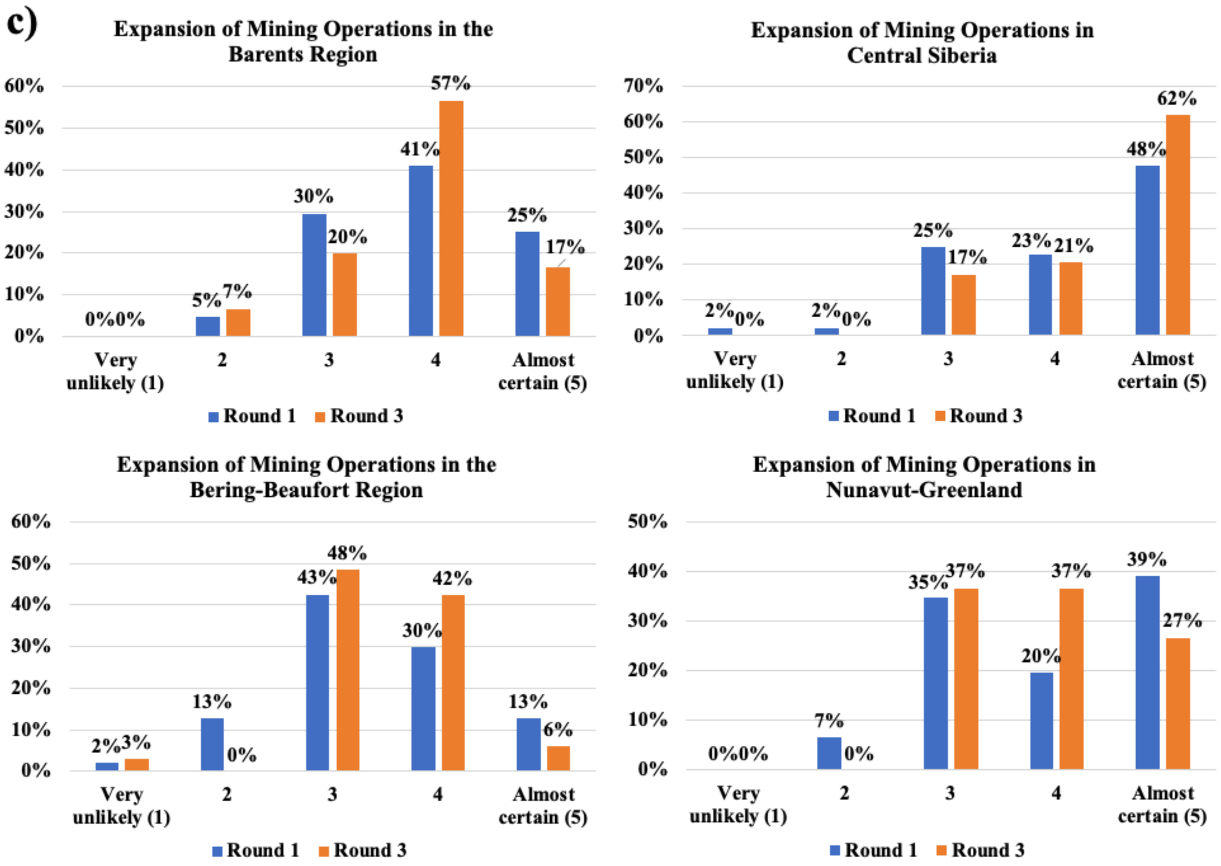

Figure 4d. Delphi results for rise in tourism

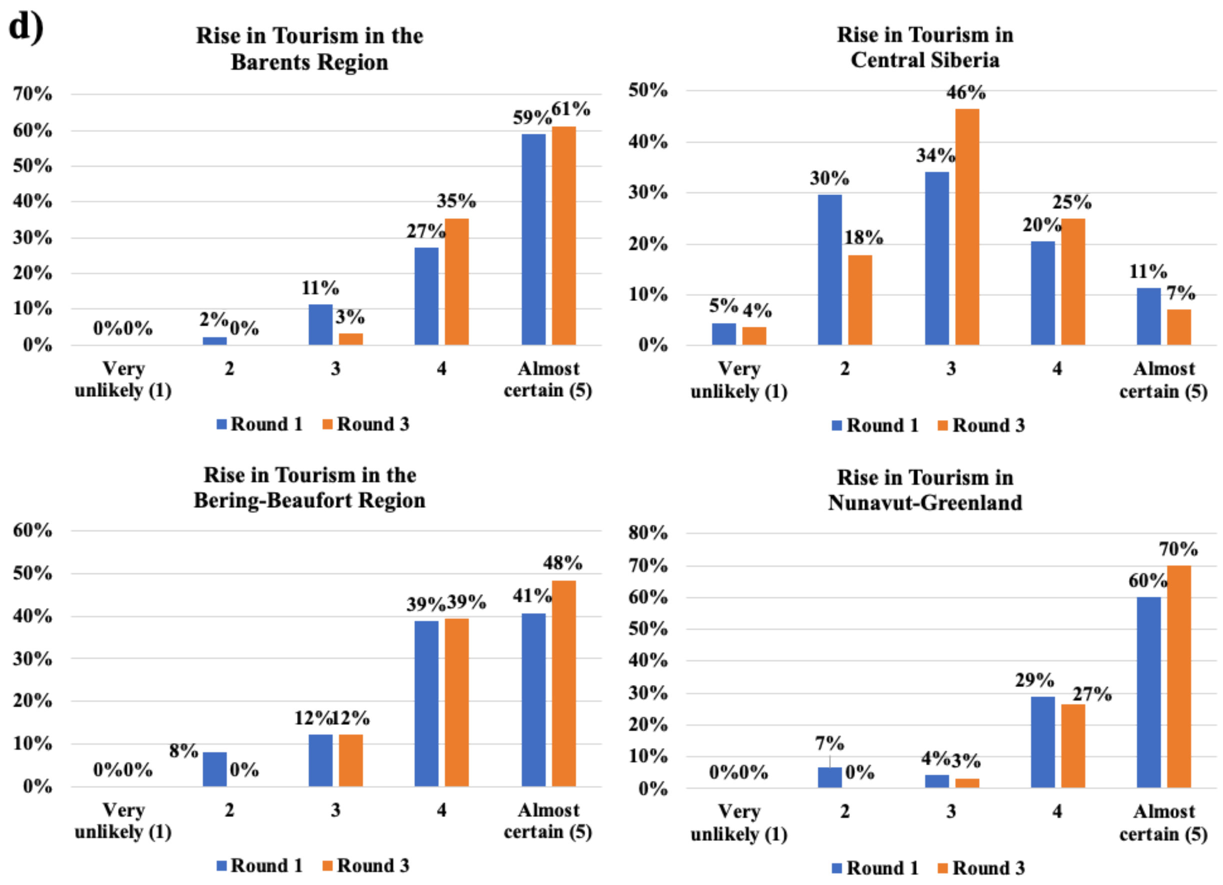

Figure 4e. Delphi results for expansion of technology sector

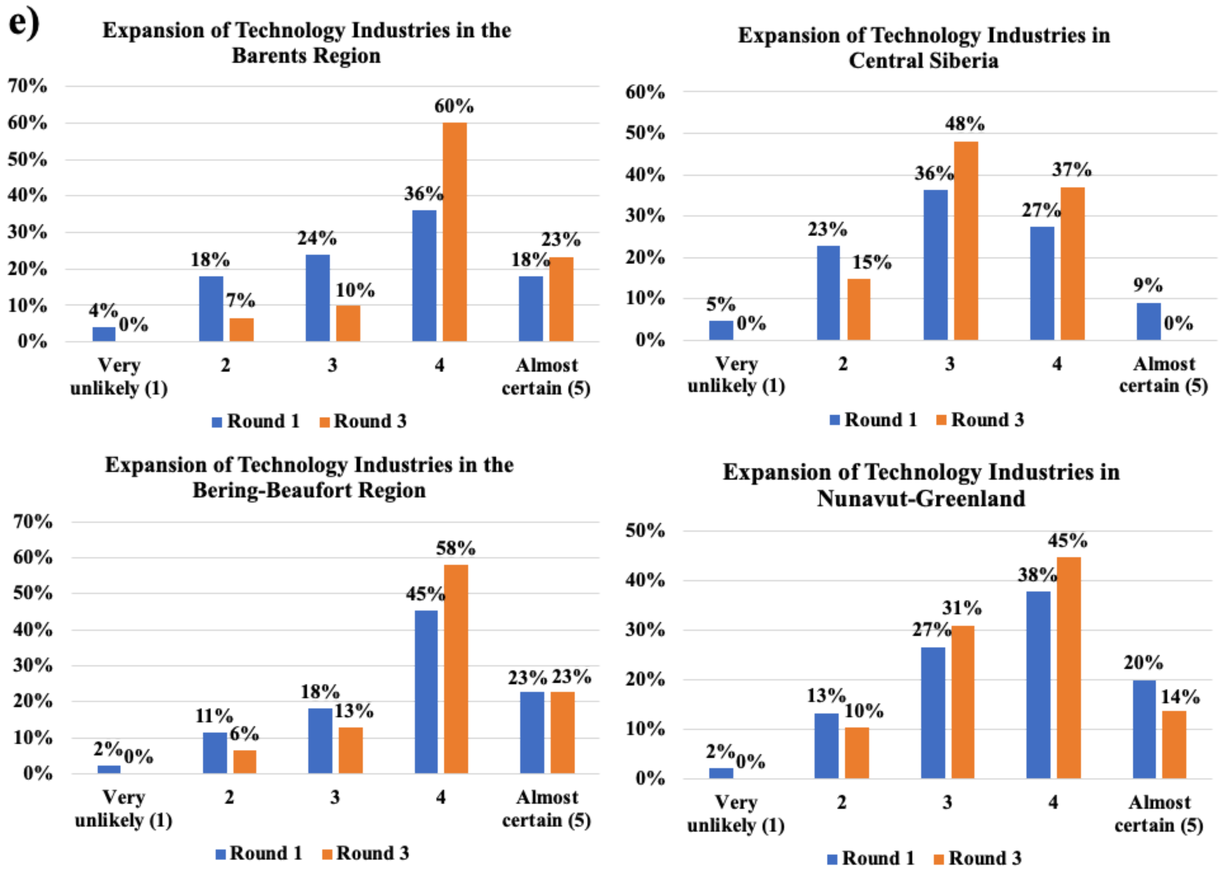

Figure 5a. Delphi results for increase in favorability towards Arctic residency

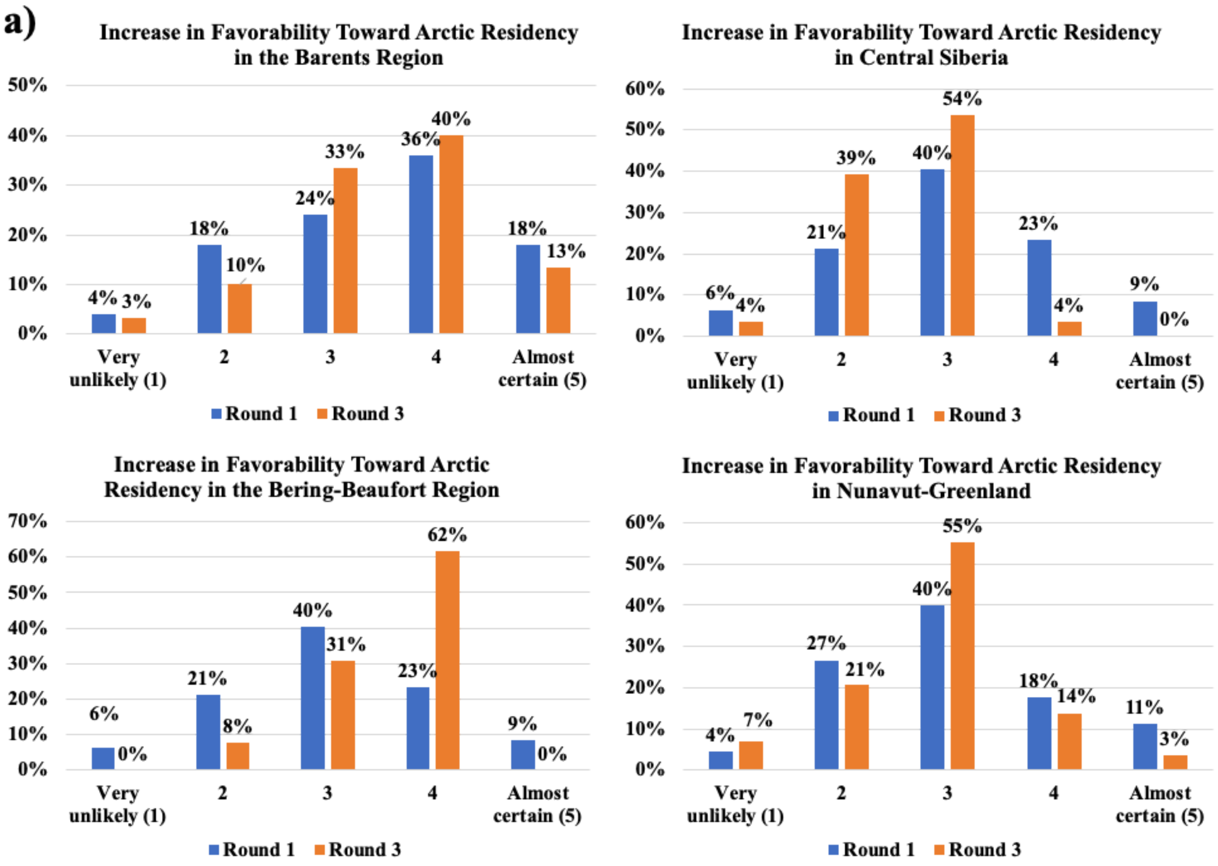

Figure 5b. Delphi results for expansion of indigenous self-determination

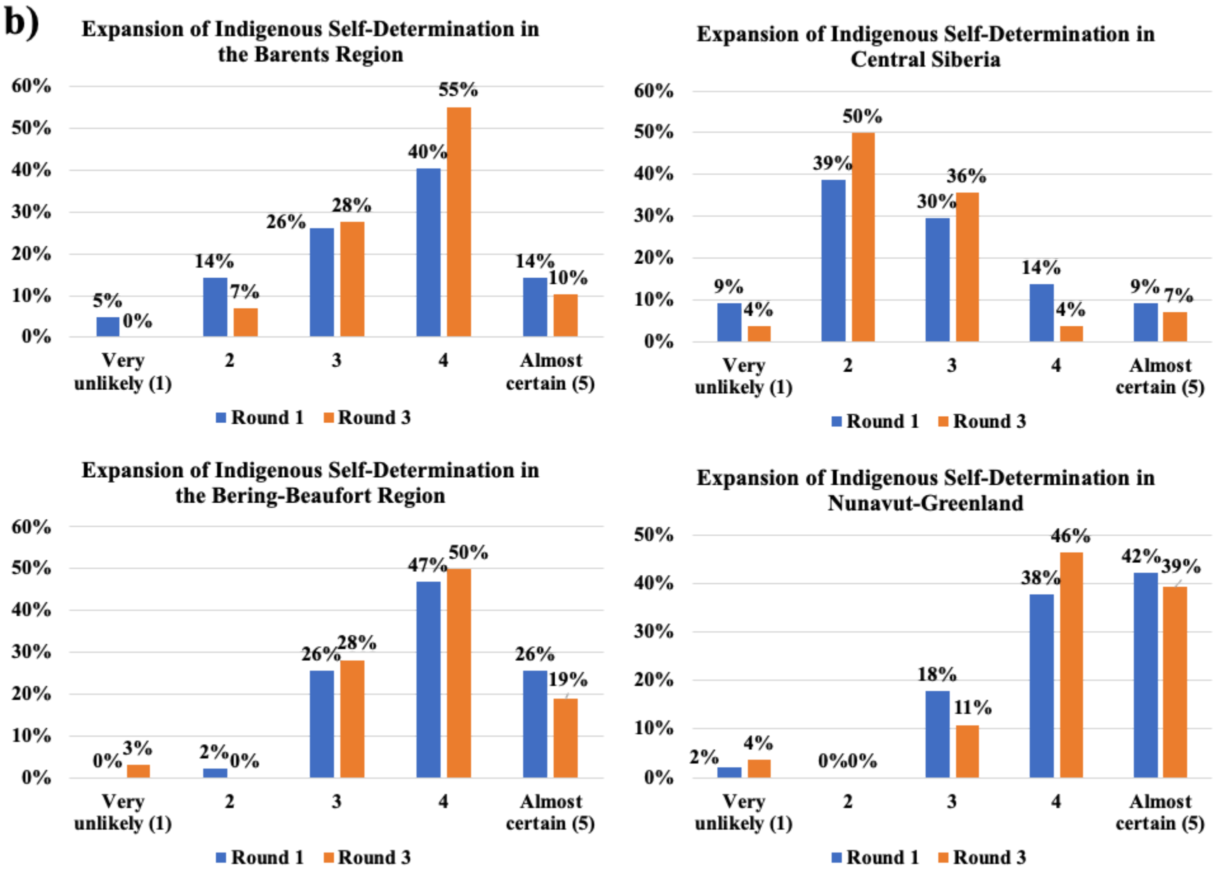

Figure 5c. Delphi results for decline in multi-stakeholder cooperation

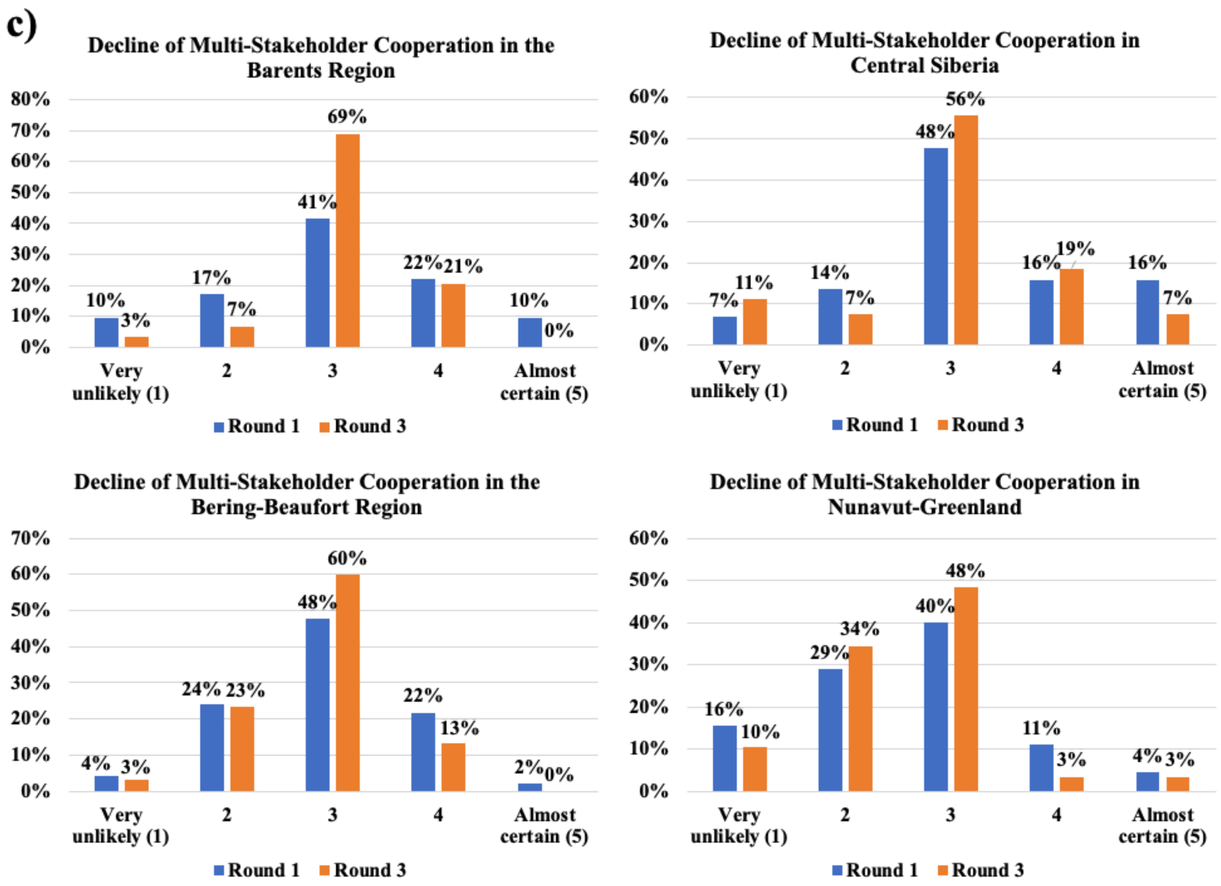

Figure 5d. Delphi results for growth of protected areas

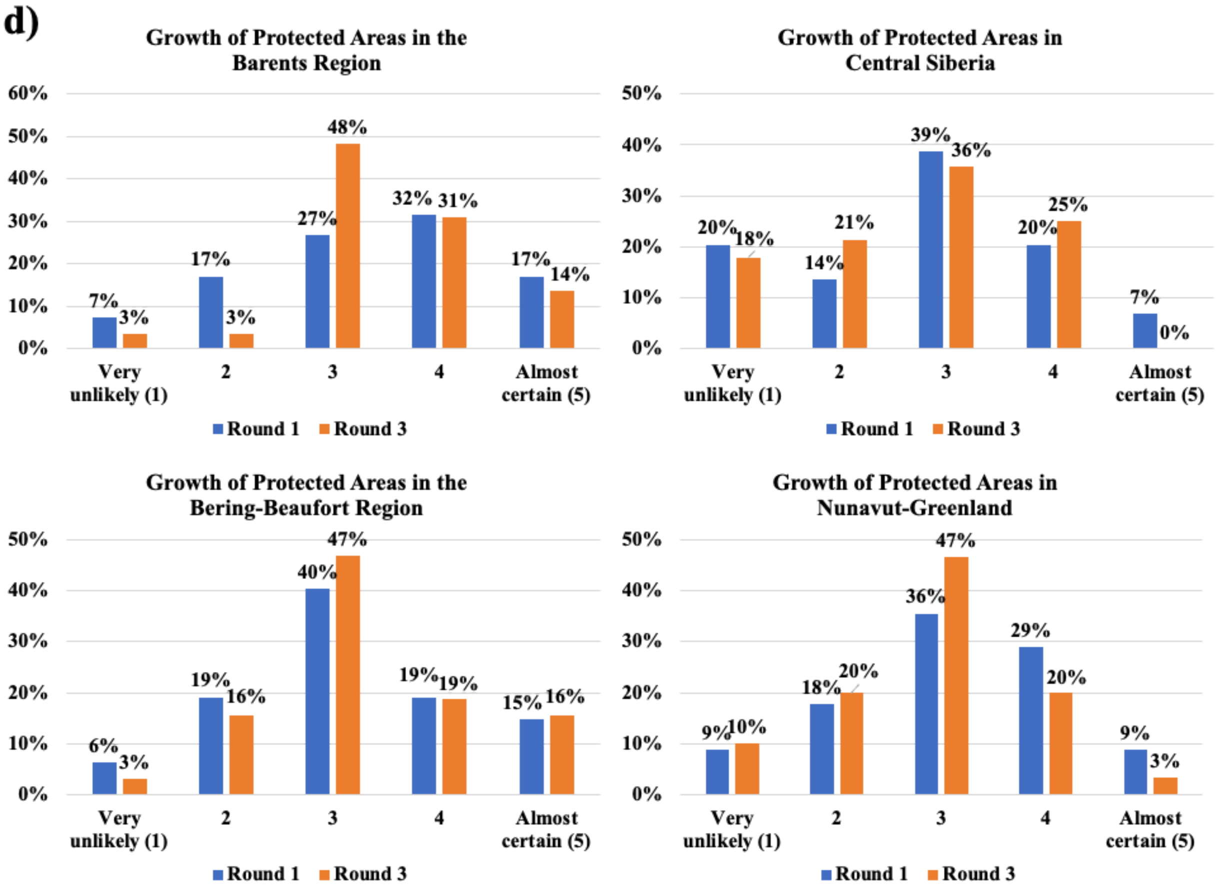

Figure 5e. Delphi results for expansion of military operations

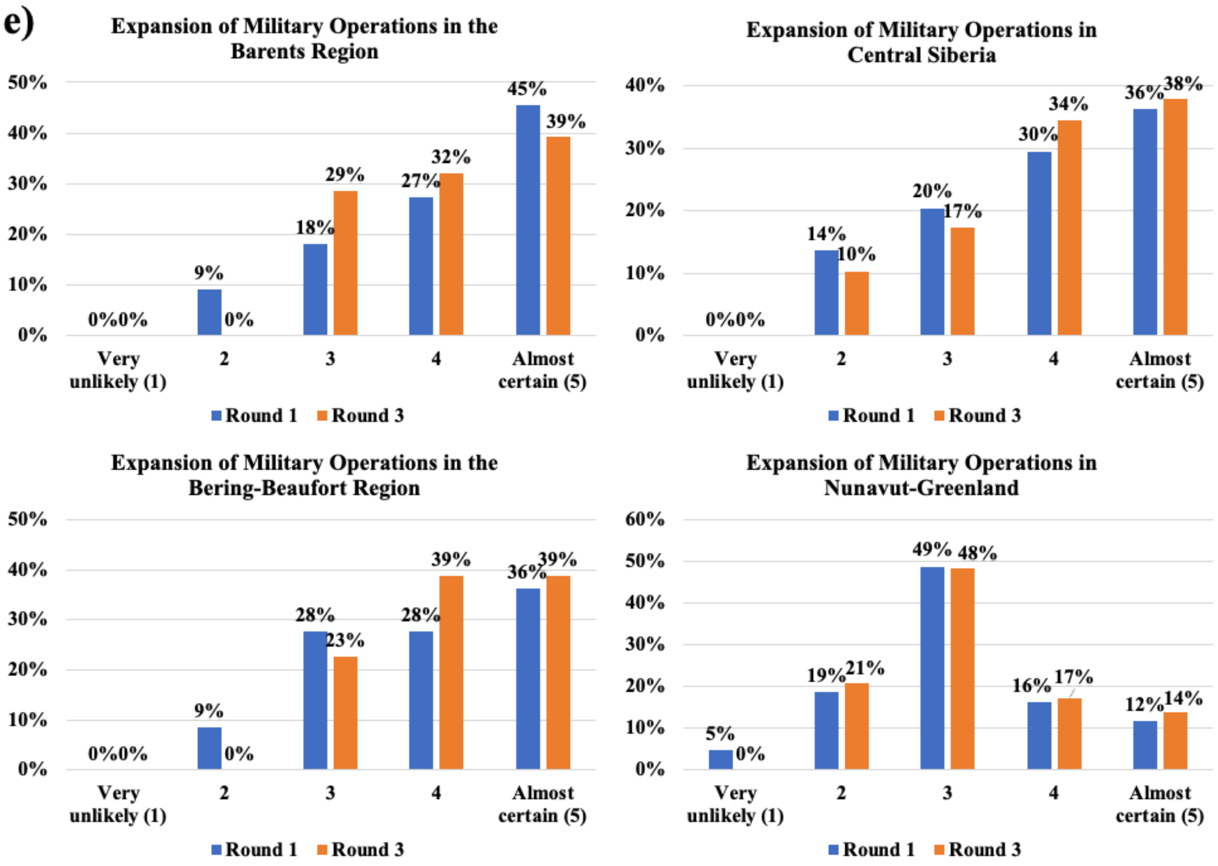

Additional information on the conduct of the Delphi exercise

The research team did not have visibility into the final demographics of the group; given the relatively small number of participants and the close-knit nature of the Arctic research community there was substantial concern about being able to link participants with their individual responses. The research team did field a short, voluntary questionnaire prior to the start of the exercise for anyone that expressed interest in participating in the exercise. Only nineteen people completed the demographic questions. Of these nineteen respondents, eleven self-identified as a woman, seven self-identified as a man, and one declined to answer; six people resided in Norway, four in Russia, five in the United States, and one each in Finland, France, India and the United Kingdom; four indicated they were a member of an Indigenous community; five indicated they are between the ages of 18 to 34, four between the ages of 35 to 49, seven between the ages of 50 to 64; two 65 or older, and one did not complete this question. For the reasons stated above, we are not able to tell how representative these demographics are of the final participant group.

Scores indicated participants' assessment of a given development outcome for a particular sub-region as follows:

1. Very unlikely
2. Unlikely
3. Possible, just as likely to happen as not
4. Likely
5. Almost certain

Participants were also encouraged to consider the following topics for context in the written comments provided alongside likelihood scores:

- **Transportation and access** including factors like the degree of road connectivity, including via winter roads, marine navigation season length and port availability, airport availability and connectivity
- **Demand for commodities** including commodities like fish, hydrocarbons, minerals, other natural products (e.g., berries, reindeer meat and hides), and timber
- **Governance, capacity, and infrastructure** including factors like the degree of Indigenous self-governance; degree of skills available in a particular location or sub-region; degree of government investment, including in healthcare; availability of schools and housing; degree of subsistence living/farming; infrastructure resilience to natural hazards
- **Technology** including factors like the availability of resource extraction or manufacturing technology, degree of broadband communications capabilities, and rate of green or smart cities technology adoption
- **Other global factors** such as geopolitics and climate change

Delphi exercise protocol

Informed Consent

Study Goals: *Navigating Convergent Pressures on Arctic Development* (CPAD) is a multi-institution, National Science Foundation (NSF) funded project that will run until 2025. It will identify key convergent and amplifying factors driving Arctic development and determine the possible trends and events that could cause tensions or conflict. Furthermore, CPAD will consider both path dependencies and emerging trends in regional decision-making, national governance, and international cooperation in the Arctic.

Your Participation in this Study: If you agree to participate, we will ask you to participate in a 3-round online expert panel:

- In Round One, you will rate a list of development factors on their importance for achieving possible development outcomes in the Arctic by 2050. You will also rate the likelihood of achieving these development outcomes in four Arctic subregions.
- In Round Two, you will discuss your Round One answers with other participants; and

- In Round Three, you will use the information from Round Two to revise your original Round One answers.

You will have approximately 7 days to complete each round. We estimate your total anticipated participation time in this study will probably be about 8 hours over 4 weeks.

Confidentiality: We will use the information you provide for research purposes only. We will not disclose your identity or information that would identify you to anyone outside of the project without your permission. We will protect the confidentiality of all your responses and discussion comments. Your responses and discussion comments will be identified by a username, rather than your given name or your email. We will destroy all information that identifies you at the end of the study. The de-identified information we collect as part of this panel may be shared with others for research purposes only.

Your Rights as a Research Participant: Your participation in this online panel is completely voluntary. You may refuse to participate, refuse to answer particular questions, and/or stop participating at any time and for any reason. RAND and its partner institutions (Brown University, Babson College, University of North Carolina, and University of Washington) will use the information you give us for research purposes only.

We have not identified any risks associated with participation in this project. Additionally, we expect that the results of this project will help inform future research initiatives aimed at understanding the implications of development in the Arctic, as well as inform sustainable, long-term infrastructure, economic, and community development plans. The results of this research may be presented at conferences and published in peer-reviewed journals.

Finally, all participants who complete all three rounds will receive a digital pass to the Arctic Frontiers 2022 Conference and will be automatically entered for a chance to win indigenous products, such as art, clothing, jewelry, or food products.*

Questions: If you have further questions concerning this online panel, please contact: Scott Stephenson at arctic-delphi@rand.org.

If you have any questions about using ExpertLens, please contact: ExpertLens Administrator at expertlens@rand.org.

If you have any questions or concerns about your rights as a participant in research, please contact **RAND's Human Subjects Protection Committee** at (866) 697-5620 or hspcinfo@rand.org. If possible, when you contact the Committee, please reference Study #2020-N0732.

By clicking on the Agree button below, you agree to participate in this online panel. You are free to withdraw from this panel at any time.

* The Official Rules for this random drawing are located here. You may enter for a chance to win the indigenous products even if you elect not to participate in this online panel. Please see the Official Rules for more information.

Introductory Text/Instructions

Welcome to Round One of the *Navigating Convergent Pressures on Arctic Development* online expert panel.

The goal of this panel is to understand what could influence development patterns and wellbeing across the Arctic by 2050.

In this panel, we ask for your opinion on the likelihood that four Arctic subregions will experience a set of possible development outcomes by 2050. We also ask you to select from a set of five factors which factor influenced your rating the most.

This panel exercise will proceed in three rounds over a period of approximately one month. Each round is expected to last for seven days. We anticipate that participation in all three rounds will take approximately 8 hours of your time.

- Round One: You will be asked to your opinion by answering a series of close-ended questions and then explain each of your responses using open-text boxes.

- Round Two: You will review your own and the group's responses from Round One. You will also discuss Round One results with other participants using an anonymous, moderated, online discussion board. We encourage logging on throughout the week to review and post comments and responses.

- Round Three: You will be able to revise your Round One responses, if you wish to do so, and share your perspective on study participation.

Please note that you may start each round at any time while the round is open. Your answers are automatically saved. You may complete the study questions in any order by clicking through the pages you wish to work on later. To move between pages, click on the navigation boxes located at the bottom of each page. Red lines above these navigation boxes will help you identify the pages that have unanswered questions in Round One and Round Three. Once you answer all questions and are on the last page, please click the "Finish" button.

If you leave the study for a period of more than 30 minutes, you should log out of ExpertLens and log back in even if you were not logged out automatically. This will help ensure that all your responses are saved properly.

ExpertLens is best viewed in Firefox, Chrome, or Safari. At this time, IE is not a supported browser for ExpertLens.

Round One

Development Outcome Likelihood Rating
In this panel, you will be asked to consider the following 10 development outcomes that the Arctic could experience by 2050:

- Accelerated hydrocarbon extraction
- Expansion of mining operations
- Rise in demand for eco- and other tourism
- Expansion and persistence of military operations
- Growth in food production (ocean and land-based)
- Decrease in permanent populations
- Expansion of technology sector
- Increased collaboration between indigenous populations and state/federal and/or private companies
- Reduced access to land and coastal/maritime areas associated with climate change mitigation
- Reduced access to land and coastal/maritime areas due to environmental/climate change related problems (e.g., coastal erosion, spread of environmental toxins)

Please rate the likelihood that the following four Arctic subregions will experience each development outcome by 2050:

- Barents region, which includes the Barents Sea, Norwegian Sea, and Greenland Sea (+ associated land areas, including Iceland and very Northwestern Russia, major cities: Arkhangelsk, Murmansk, Rovaniemi, Inari, Kirkenes, Tromsø, Bodø)
- Central Siberia, which includes the Kara Sea, Laptev Sea, and East Siberian Sea (+ associated land areas, major cities: Salekhard, Dudinka, Yakutsk)
- Bering/Beaufort region, which includes the Bering Sea, Beaufort Sea (+ associated land areas including the Chukotka Autonomous Okrug, all of coastal Alaska, and Yukon/NWT in Canada, major cities: Anadyr, Fairbanks, Whitehorse, Yellowknife)
- Nunavut-Greenland, which includes the Canadian Archipelago and Baffin Bay (+ associated land areas in Nunavut and western Greenland, major cities: Iqaluit, Rankin Inlet, Nuuk, Sisimiut, Ilulissat)

Rating Scale

You will use the following 5-point likelihood scale, where:

- A score of 1 indicates a development outcome is very unlikely for a given region.
- A score of 2 indicates a development outcome is unlikely for a given region, meaning it is less likely to happen than not.
- A score of 3 indicates a development outcome is possible for a given region, meaning it is just as likely to happen as not.
- A score of 4 indicates a development outcome is likely for a given region, meaning it is more likely to happen than not.
- A score of 5 indicates a development outcome is almost certain for a given region, meaning you would be surprised if it did not happen.

We encourage you to consider the full scale when rating the likelihood of the development outcomes. Please note that there is no objectively “correct” answer.

When rating the likelihood each region might experience, you are asked to consider the following five factor categories that could potentially affect Arctic development by 2050:

- Transportation and access, which includes factors like the degree of road connectivity, including via winter roads, marine navigation season length and port availability, airport availability and connectivity
- Demand for commodities, which includes commodities like fish, hydrocarbons, minerals, other natural products (e.g., berries, reindeer meat and hides), and timber
- Local governance, capacity and infrastructure, which includes factors like the degree of Indigenous self-governance; degree of locally available skills; degree of national government investment, including in healthcare; degree of subsistence living/farming; infrastructure resilience to natural hazards
- Technology, which includes factors like the availability of resource extraction or manufacturing technology, degree of broadband communications penetration, and rate of green or smart cities technology adoption
- Other global factors, which includes factors like political constraints on maritime or economic access, climate stabilization and mitigation capacity, and degree of foreign direct investment

Explaining Your Ratings

In addition to providing a numeric response to each question, we ask that you explain your rating using the open text boxes, specifically which factors influenced your rating the most. Your Round One ratings and explanations will be anonymized and shown to all study participants in Rounds Two and Three. For this reason, it is important that you provide a detailed explanation of your responses. This information may help other participants interpret your responses.

Round Two

Welcome to Round Two of the *Navigating Convergent Pressures on Arctic Development* online expert panel.

In Round One, you offered your expert opinion on how likely ten development outcomes are to happen in four Arctic subregions and which factor category influenced your rating the most. In Round Two, we ask that you review Round One results and discuss them with other participants.

Review Round One Results

On the following pages, you will see how your Round One responses compare to those of other participants. Each Round One question will have its own chart and summary of participant comments. You will see the following information:

- Yellow bars on the chart that show the percentage of participants who provided that answer in Round One (hover over each bar to view the number of participants).
- Red dot on the chart that indicates your own Round One answer (if you do not see a red dot, you did not answer this question in Round One).
- Blue line on the chart that shows the group median (half of participants chose an answer that was above this value; the other half chose an answer that was equal to or below it. Hover over the blue line to see the value of the median).
- Color-coded decision below the chart that shows whether participants agreed with each other and whether the overall rating was of low, uncertain, or high importance.
- Summary of participants' comments next to the chart that can help you better understand what factors affected the ratings of participants who felt that this recommendation was of low, uncertain, or high appropriateness or necessity.
- Individual participants' comments below the comments' summary that can help you better understand why other participants chose a particular response. You will need to click on the link to view these comments.

2) Discuss Round One results: Once you review Round One results, please discuss them with other participants. You can share your thoughts in three ways:

- Comment on a summary of participants' responses to a particular question by either starting a new discussion thread or responding to an existing comment.
- Agree or disagree with a specific comment made by other participants in response to a Round One question by clicking on [thumbs up or down]. You will need to click on the link "View Participants' Round One Comments" to see individual comments from Round One.
- Participate in a discussion at the top of each page by posting more general comments that cut across the care statement presented on that page.

Please note that you have to click the "Submit" button located next to each comment box so that your comment can be saved.

Round Two is designed to help you investigate any aspect of the responses you think might be important. For example, if your response to a particular question is very different from others' responses, you may want to further explain your perspective. You may want to ask other participants to clarify the reasons behind their responses. You can also engage with our discussion moderators if you have questions about a particular statement or if something is not clear.

We realize that you may not have direct experiences/expertise with every region in the Arctic or with all of the development outcomes or factors. In this round, you will be able to learn directly from experts who may have direct knowledge. Feel free to ask them specific questions about each development outcome or Arctic subregion if you think this information can help you better understand the perspectives of other participants or provide a more informed response to our questions in Round Three.

We would like to encourage a free and open exchange of ideas during this online discussion. That is why we are not revealing the identities of panel members. However, you will be able to see all comments made by a given participant by looking at their participant IDs.

Because participants will be contributing comments at different times, we encourage you to check back regularly throughout Round Two. Your identity will remain concealed from all other participants throughout this discussion.

If you leave the study for a period of more than 30 minutes, you should log out of ExpertLens and log back in even if you were not logged out automatically. This will help ensure that all your responses are saved properly.

ExpertLens is best viewed in Firefox, Chrome, or Safari. At this time, IE is not a supported browser for ExpertLens.

Round Three

Welcome to Round Three of the *Navigating Convergent Pressures on Arctic Development* online expert panel.

In this round, we ask that you review the Round Two discussions and revise your Round One responses if needed. Even if your Round One responses have not changed, we ask that you still re-enter them. If your answer to a particular question has not changed, click on “Use my answer from Round One” and your numeric response from Round One will be automatically entered as your Round Three response. We encourage you to use the text box to explain why your answer has or has not changed. While you can read Round Two discussion topics, you will no longer be able to respond to them.

1) Rating Criteria: As a reminder, we ask that you rate the likelihood that the following four Arctic subregions will experience the following 10 development outcomes by 2050. We also ask

you to explain your rating in an open text box specifically what factor influenced your rating the most.

Development Outcomes

- Accelerated hydrocarbon extraction
- Expansion of mining operations
- Rise in demand for eco- and other tourism
- Expansion and persistence of military operations
- Growth in food production (ocean and land-based)
- Decrease in permanent populations
- Expansion of technology sector
- Increased collaboration between indigenous populations and state/federal and/or private companies
- Reduced access to land and coastal/maritime areas associated with climate change mitigation
- Reduced access to land and coastal/maritime areas due to environmental/climate change related problems (e.g., coastal erosion, spread of environmental toxins)

Arctic Subregions

- Barents region, which includes the Barents Sea, Norwegian Sea, and Greenland Sea (+ associated land areas, including Iceland and very Northwestern Russia, major cities: Arkhangelsk, Murmansk, Rovaniemi, Inari, Kirkenes, Tromsø, Bodø)
- Central Siberia, which includes the Kara Sea, Laptev Sea, and East Siberian Sea (+ associated land areas, major cities: Salekhard, Dudinka, Yakutsk)
- Bering/Beaufort region, which includes the Bering Sea, Beaufort Sea (+ associated land areas including the Chukotka Autonomous Okrug, all of coastal Alaska, and Yukon/NWT in Canada, major cities: Anadyr, Fairbanks, Whitehorse, Yellowknife)
- Nunavut-Greenland, which includes the Canadian Archipelago and Baffin Bay (+ associated land areas in Nunavut and western Greenland, major cities: Iqaluit, Rankin Inlet, Nuuk, Sisimiut, Ilulissat)

2) Rating Scale:

To rate the likelihood that a subregion will experience a given development outcome, you will use the following likelihood scale, where:

- A score of 1 indicates a development outcome is very unlikely for a given region.
- A score of 2 indicates a development outcome is unlikely for a given region, meaning it is less likely to happen than not.
- A score of 3 indicates a development outcome is possible for a given region, meaning it is just as likely to happen as not.
- A score of 4 indicates a development outcome is likely for a given region, meaning it is more likely to happen than not.
- A score of 5 indicates a development outcome is almost certain for a given region, meaning you would be surprised if it did not happen.

We encourage you to consider the full scale when rating the likelihood of the development outcomes. We also ask that you provide comments using open text boxes so that we can better understand your thoughts and reasons for changing or not changing your original responses. Please note that there is no objectively “correct” answer.

3) Page navigation: To move between pages, click on the purple navigation boxes located at the bottom of each page. Red lines above them will help you identify the pages that have unanswered questions.

On the last page, you will have an opportunity to share your experiences with our online platform. Once you answer all questions and are on the last page, please click the "Finish" button.

If you leave the study for a period of more than 30 minutes, you should log out of ExpertLens and log back in even if you were not logged out automatically. This will help ensure that all your responses are saved properly.

ExpertLens is best viewed in Firefox, Chrome, or Safari. At this time, IE is not a supported browser for ExpertLens.

Subgroups and Question

Subgroup title: Barents Region

On this page, you are asked to rate the likelihood that the Barents Region will experience 10 possible development outcomes and explain your rating with the specific factor that influenced your rating the most.

The Barents Region includes the Barents Sea, Norwegian Sea, and Greenland Sea (+ associated land areas, including Iceland and very Northwestern Russia, major cities: Arkhangelsk, Murmansk, Rovaniemi, Inari, Kirkenes, Tromsø, Bodø)

The 10 development outcomes are:

- Accelerated hydrocarbon extraction
- Expansion of mining operations
- Rise in demand for eco- and other tourism
- Expansion and persistence of military operations
- Growth in food production (ocean and land-based)
- Decrease in permanent populations
- Expansion of technology sector
- Increased collaboration between indigenous populations and state/federal and/or private companies
- Reduced access to land and coastal/maritime areas associated with climate change mitigation
- Reduced access to land and coastal/maritime areas due to environmental/climate change related problems (e.g., coastal erosion, spread of environmental toxins)

The five factor categories are:

- Transportation and access, which includes factors like the degree of road connectivity, including via winter roads, marine navigation season length and port availability, airport availability and connectivity
- Demand for commodities, which includes commodities like fish, hydrocarbons, minerals, other natural products (e.g., berries, reindeer meat and hides), and timber
- Local governance, capacity and infrastructure, which includes factors like the degree of Indigenous self-governance; degree of locally available skills; degree of national government investment, including in healthcare; degree of subsistence living/farming; infrastructure resilience to natural hazards
- Technology, which includes factors like the availability of resource extraction or manufacturing technology, degree of broadband communications penetration, and rate of green or smart cities technology adoption
- Other global factors, which includes factors like political constraints on maritime or economic access, climate stabilization and mitigation capacity, and degree of foreign direct investment

Question 1: How likely is it that the Barents region will experience accelerated hydrocarbon extraction by 2050?

Question 2: How likely is it that the Barents region will experience expansion of mining operations by 2050

Question 3: How likely is it that the Barents region will experience a rise in demand for eco- or other tourism by 2050?

Question 4: How likely is it that the Barents region will experience expansion and persistence of military operations by 2050?

Question 5: How likely is it that the Barents region will experience growth in food production (ocean and land-based) by 2050?

Question 6: How likely is it that the Barents region will experience a decrease in permanent populations by 2050?

Question 7: How likely is it that the Barents region will experience expansion of the technology sector by 2050?

Question 8: How likely is it that the Barents region will experience increased collaboration between indigenous populations and state/federal and/or private companies by 2050?

Question 9: How likely is it that the Barents region will experience reduced access to land and coastal/maritime areas associated with climate change mitigation by 2050?

Question 10: How likely is it that the Barents region will experience reduced access to land and coastal/maritime areas due to environmental/climate change related problems (e.g., coastal erosion, spread of environmental toxins) by 2050?

Subgroup title: Central Siberia

On this page, you are asked to rate the likelihood that Central Siberia will experience 10 possible development outcomes and explain your rating with the specific factor that influenced your rating the most.

The Barents Region includes the Kara Sea, Laptev Sea, and East Siberian Sea (+ associated land areas, major cities: Salekhard, Dudinka, Yakutsk)

The 10 development outcomes are:

- Accelerated hydrocarbon extraction
- Expansion of mining operations
- Rise in demand for eco- and other tourism
- Expansion and persistence of military operations
- Growth in food production (ocean and land-based)
- Decrease in permanent populations
- Expansion of technology sector
- Increased collaboration between indigenous populations and state/federal and/or private companies
- Reduced access to land and coastal/maritime areas associated with climate change mitigation
- Reduced access to land and coastal/maritime areas due to environmental/climate change related problems (e.g., coastal erosion, spread of environmental toxins)

The five factor categories are:

- Transportation and access, which includes factors like the degree of road connectivity, including via winter roads, marine navigation season length and port availability, airport availability and connectivity
- Demand for commodities, which includes commodities like fish, hydrocarbons, minerals, other natural products (e.g., berries, reindeer meat and hides), and timber
- Local governance, capacity and infrastructure, which includes factors like the degree of Indigenous self-governance; degree of locally available skills; degree of national government investment, including in healthcare; degree of subsistence living/farming; infrastructure resilience to natural hazards
- Technology, which includes factors like the availability of resource extraction or manufacturing technology, degree of broadband communications penetration, and rate of green or smart cities technology adoption
- Other global factors, which includes factors like political constraints on maritime or economic access, climate stabilization and mitigation capacity, and degree of foreign direct investment

Question 1: How likely is it that the Central Siberia will experience accelerated hydrocarbon extraction by 2050*?*

Question 2: How likely is it that the Central Siberia will experience expansion of mining operations by 2050?

Question 3: How likely is it that the Central Siberia will experience a rise in demand for eco- or other tourism by 2050?

Question 4: How likely is it that the Central Siberia will experience expansion and persistence of military operations by 2050?

Question 5: How likely is it that the Central Siberia will experience growth in food production (ocean and land-based) by 2050?

Question 6: How likely is it that the Central Siberia will experience a decrease in permanent populations by 2050?

Question 7: How likely is it that the Central Siberia will experience expansion of the technology sector by 2050?

Question 8: How likely is it that the Central Siberia will experience increased collaboration between indigenous populations and state/federal and/or private companies by 2050?

Question 9: How likely is it that the Central Siberia will experience reduced access to land and coastal/maritime areas associated with climate change mitigation by 2050?

Question 10: How likely is it that the Central Siberia will experience reduced access to land and coastal/maritime areas due to environmental/climate change related problems (e.g., coastal erosion, spread of environmental toxins) by 2050?

Subgroup title: Bering/Beaufort

On this page, you are asked to rate the likelihood that Bering/Beaufort region will experience 10 possible development outcomes and explain your rating with the specific factor that influenced your rating the most.

The Bering/Beaufort region includes the Bering Sea, Beaufort Sea (+ associated land areas including the Chukotka Autonomous Okrug, all of coastal Alaska, and Yukon/NWT in Canada, major cities: Anadyr, Fairbanks, Whitehorse, Yellowknife)
The 10 development outcomes are:

- Accelerated hydrocarbon extraction
- Expansion of mining operations
- Rise in demand for eco- and other tourism
- Expansion and persistence of military operations
- Growth in food production (ocean and land-based)
- Decrease in permanent populations
- Expansion of technology sector
- Increased collaboration between indigenous populations and state/federal and/or private companies
- Reduced access to land and coastal/maritime areas associated with climate change mitigation
- Reduced access to land and coastal/maritime areas due to environmental/climate change related problems (e.g., coastal erosion, spread of environmental toxins)

The five factor categories are:

- Transportation and access, which includes factors like the degree of road connectivity, including via winter roads, marine navigation season length and port availability, airport availability and connectivity
- Demand for commodities, which includes commodities like fish, hydrocarbons, minerals, other natural products (e.g., berries, reindeer meat and hides), and timber
- Local governance, capacity and infrastructure, which includes factors like the degree of Indigenous self-governance; degree of locally available skills; degree of national government investment, including in healthcare; degree of subsistence living/farming; infrastructure resilience to natural hazards
- Technology, which includes factors like the availability of resource extraction or manufacturing technology, degree of broadband communications penetration, and rate of green or smart cities technology adoption
- Other global factors, which includes factors like political constraints on maritime or economic access, climate stabilization and mitigation capacity, and degree of foreign direct investment

Question 1: How likely is it that the Bering/Beaufort region will experience accelerated hydrocarbon extraction by 2050?

Question 2: How likely is it that the Bering/Beaufort region will experience expansion of mining operations by 2050?

Question 3: How likely is it that the Bering/Beaufort region will experience a rise in demand for eco- or other tourism by 2050?

Question 4: How likely is it that the Bering/Beaufort region will experience expansion and persistence of military operations by 2050?

Question 5: How likely is it that the Bering/Beaufort region will experience growth in food production (ocean and land-based) by 2050?

Question 6: How likely is it that the Bering/Beaufort region will experience a decrease in permanent populations by 2050?

Question 7: How likely is it that the Bering/Beaufort region will experience expansion of the technology sector by 2050?

Question 8: How likely is it that the Bering/Beaufort region will experience increased collaboration between indigenous populations and state/federal and/or private companies by 2050?

Question 9: How likely is it that the Bering/Beaufort region will experience reduced access to land and coastal/maritime areas associated with climate change mitigation by 2050?

Question 10: How likely is it that the Bering/Beaufort region will experience reduced access to land and coastal/maritime areas due to environmental/climate change related problems (e.g., coastal erosion, spread of environmental toxins) by 2050?

Subgroup title: Nunavut-Greenland

On this page, you are asked to rate the likelihood that Nunavut-Greenland region will experience 10 possible development outcomes and explain your rating with the specific factor that influenced your rating the most.

The Nunavut-Greenland region includes the Canadian Archipelago and Baffin Bay (+ associated land areas in Nunavut and western Greenland, major cities: Iqaluit, Rankin Inlet, Nuuk, Sisimiut, Ilulissat)
The 10 development outcomes are:

- Accelerated hydrocarbon extraction
- Expansion of mining operations
- Rise in demand for eco- and other tourism
- Expansion and persistence of military operations
- Growth in food production (ocean and land-based)
- Decrease in permanent populations
- Expansion of technology sector
- Increased collaboration between indigenous populations and state/federal and/or private companies
- Reduced access to land and coastal/maritime areas associated with climate change mitigation
- Reduced access to land and coastal/maritime areas due to environmental/climate change related problems (e.g., coastal erosion, spread of environmental toxins)

The five factor categories are:

- Transportation and access, which includes factors like the degree of road connectivity, including via winter roads, marine navigation season length and port availability, airport availability and connectivity
- Demand for commodities, which includes commodities like fish, hydrocarbons, minerals, other natural products (e.g., berries, reindeer meat and hides), and timber
- Local governance, capacity and infrastructure, which includes factors like the degree of Indigenous self-governance; degree of locally available skills; degree of national government investment, including in healthcare; degree of subsistence living/farming; infrastructure resilience to natural hazards
- Technology, which includes factors like the availability of resource extraction or manufacturing technology, degree of broadband communications penetration, and rate of green or smart cities technology adoption
- Other global factors, which includes factors like political constraints on maritime or economic access, climate stabilization and mitigation capacity, and degree of foreign direct investment

Question 1: How likely is it that the Nunavut-Greenland region will experience accelerated hydrocarbon extraction by 2050?

Question 2: How likely is it that the Nunavut-Greenland region will experience expansion of mining operations by 2050

Question 3: How likely is it that the Nunavut-Greenland region will experience a rise in demand for eco- or other tourism by 2050?

Question 4: How likely is it that the Nunavut-Greenland region will experience expansion and persistence of military operations by 2050?

Question 5: How likely is it that the Nunavut-Greenland region will experience growth in food production (ocean and land-based) by 2050?

Question 6: How likely is it that the Nunavut-Greenland region will experience a decrease in permanent populations by 2050?

Question 7: How likely is it that the Nunavut-Greenland region will experience expansion of the technology sector by 2050?

Question 8: How likely is it that the Nunavut-Greenland region will experience increased collaboration between indigenous populations and state/federal and/or private companies by 2050?

Question 9: How likely is it that the Nunavut-Greenland region will experience reduced access to land and coastal/maritime areas associated with climate change mitigation by 2050?

Question 10: How likely is it that the Nunavut-Greenland region will experience reduced access to land and coastal/maritime areas due to environmental/climate change related problems (e.g., coastal erosion, spread of environmental toxins) by 2050?
